# When One Size Does Not Fit All: A Simple Statistical Method to Deal with Across-Individual Variations of Effects

**DOI:** 10.1371/journal.pone.0039059

**Published:** 2012-06-18

**Authors:** Philippe Vindras, Michel Desmurget, Pierre Baraduc

**Affiliations:** Cognitive Neuroscience Center, CNRS UMR 5229, Bron, France; Research and Development Corporation, United States of America

## Abstract

In science, it is a common experience to discover that although the investigated effect is very clear in some individuals, statistical tests are not significant because the effect is null or even opposite in other individuals. Indeed, t-tests, Anovas and linear regressions compare the average effect with respect to its inter-individual variability, so that they can fail to evidence a factor that has a high effect in many individuals (with respect to the intra-individual variability). In such paradoxical situations, statistical tools are at odds with the researcher’s aim to uncover any factor that affects individual behavior, and not only those with stereotypical effects. In order to go beyond the reductive and sometimes illusory description of the average behavior, we propose a simple statistical method: applying a Kolmogorov-Smirnov test to assess whether the distribution of *p*-values provided by individual tests is significantly biased towards zero. Using Monte-Carlo studies, we assess the power of this two-step procedure with respect to RM Anova and multilevel mixed-effect analyses, and probe its robustness when individual data violate the assumption of normality and homoscedasticity. We find that the method is powerful and robust even with small sample sizes for which multilevel methods reach their limits. In contrast to existing methods for combining *p*-values, the Kolmogorov-Smirnov test has unique resistance to outlier individuals: it cannot yield significance based on a high effect in one or two exceptional individuals, which allows drawing valid population inferences. The simplicity and ease of use of our method facilitates the identification of factors that would otherwise be overlooked because they affect individual behavior in significant but variable ways, and its power and reliability with small sample sizes (<30–50 individuals) suggest it as a tool of choice in exploratory studies.

## Introduction

### An Example of Individually Variable Effect

Sometimes reality is more complex than common effects found in all individuals. Consider for example an experiment on visually-guided reaching, designed to test the accuracy of human subjects. Ten volunteers are asked to reach towards visual targets with their unseen hand. The design involves repeated measures: each subject performs a similar series of movements of various distances and directions. The ratios between movement distance and actual target distance (taken as a measure of individual performance) are subjected to a paired t-test. The outcome is far from significance, because 4 out of 10 subjects systematically overshot the targets, whereas 2 others systematically undershot them. Although individual tests show that the inaccuracy is significant for 6 subjects, the experimenter has no choice but to conclude that there is no effect. Later, another experimenter interested in this apparently unexplored issue is luckier with his subjects – or finds good reasons to discard one or two outliers. He eventually reports that human subjects tend to overshoot targets when reaching without vision of the hand – or perhaps the opposite. Although the epilogue of this story is fictitious, the rest is real, and may well remind the reader of a similar situation in his or her research.

The true story ended differently since the first experimenter (actually, two of us, [1]) assessed whether a set of individual tests was globally significant, using a simple method. The result supported the general inference that the human motor system uses a visuo-motor gain to plan hand movements. This article generalizes this method to all experimental designs with repeated-measures, and thoroughly analyzes its power and reliability.

### The Problem of the Publication Bias Towards Stereotypical Effects

The example above points to a mismatch between usual statistical tools and scientific aims - the question is often whether a factor affects individual behavior, not whether it has a stereotypical effect. Research often drifts towards the latter question because of a lack of adequate tools to answer the former. As we show below, the problem is far from being circumscribed to a specific test or scientific field. The statistically savvy experimenter may resort to complex methods that can evidence individually variable effects, especially using covariates and carrying out multilevel mixed-effects analyses. However, these and others methods have several drawbacks that limit their use. Instead, we propose here a much simpler but usually as effective statistical procedure that answers the researcher’s original question. We first need to realize that the difficulty raised in the example above concerns all statistical methods based on the General(ized) Linear Model. These tests have optimal power when individuals behave identically, i.e. when the apparent inter-individual variability only results from intra-individual variability. When there exists genuine, idiosyncratic variations in the effect of a factor, the power of these tests tends towards zero as inter-individual variability increases. In the extreme, the effect of a factor can be significant for every individual (compared to intra-individual variability) while Student and Fisher tests yield a probability close to one if the population average is small enough. In such a case, the experimenter has a wrong tool for a right question – or a right tool for a wrong question.

In statistical jargon, usual procedures assess the *null average hypothesis* (that the average effect is zero), rather than the *global null hypothesis* that there is no effect in any individual (the second is also called *conjunction of null hypotheses* [2], [3] or *combined null hypothesis* [4]). This problem affects virtually all research in life and social sciences. Indeed, all objects investigated in social and life sciences are complex individual systems or subsystems. As such, their behavior depends on multiple interacting components, and they are all likely to display idiosyncratic variations of experimental factor effects. Factor effects with small inter-individual variability are much more likely to be reported than those with large variability, although the latter would shed more light on the underlying systems. In our aforementioned study, the systematic inaccuracies revealed highly significant (F tests, p<0.001 for 6 out of 10 subjects). However, the average gain error was not significantly different from 0 (Wilcoxon and Student tests, p>.25): this apparently unbiased performance did not correctly describe the behavior of 60% of the sample!

### A Simple Solution

There are presently different methods for dealing with inter-individual variability of factor effects, often by assessing the *global null hypothesis.* Multilevel mixed effects modeling is the first of them, and tends to become standard. A second solution is including covariates in an analysis of covariance (Ancova). When repeated-measures (RM) Anovas are appropriate, a third way to evidence significant but variable effects is by testing interactions between subjects and fixed factors with respect to the pooled intra-individual variability. Last, a fourth procedure has been proposed for fMRI and microarray studies [2], [3], [5]–[7] as well as social data [4]; it consists in carrying out individual fixed-effects tests such as Anovas, and then assessing whether the set of individual *p*-values is significantly biased towards zero using meta-analytic methods for combining *p*-values [8]–[10]. However, as will be shown below, each of these four methods has specific drawbacks that limit their use.

The new method we propose is akin to this last procedure. It consists in carrying out individual tests, and then assessing whether the set of individual *p*-values is biased towards zero using the Kolmogorov-Smirnov (KS) distribution test. Indeed, the *global null hypothesis* implies that the *p*-values yielded by individual tests are uniformly distributed between 0 and 1. As the one-sample Kolmogorov-Smirnov test assesses whether a sample is likely to be drawn from a theoretical distribution, the unilateral one-sample Kolmogorov-Smirnov (UKS) test will assess the likelihood of excess of small *p*-values in samples randomly drawn from the uniform distribution between 0 and 1, and thus answer our question. In the previous example on manual pointing, the UKS test applied to the outcomes of individuals tests rejected the hypothesis that humans do not make systematic movement amplitude errors (T_K_ = 0.676, p<.0001).

Compared to the existing methods for assessing the global null hypothesis, the UKS test procedure has four desirable qualities. First, it is of simple use. As it requires much less statistical expertise than multilevel mixed-effects analyses, anyone interested in testing the global null hypothesis can employ it. Second, the procedure is practically assumption-free. As tests are individual, there is no need for homoscedasticity across subjects. For the same reason, individual effects need not have a Gaussian distribution (non-parametric tests like the Kruskal-Wallis or Spearman’s rank correlation test can be used in such a case). Thus, our procedure can work in circumstances where RM Anovas and Ancovas would be impossible and multilevel analyses particularly complex. Third, this method has a rare quality: resistance. Indeed, using a distribution test makes the procedure more robust with respect to outlying individuals that any of the present methods (see **Results S1**). RM Anovas or meta-analytic methods [8]–[10] for combining *p*-values are all highly sensitive to outliers and can conclude to a significant effect because of a single individual. Fourth, our procedure works well with small samples, which makes it attractive with respect to multilevel mixed-effects analyses that need at least 30 to 50 individuals to yield accurate estimates in regressions [11]–[14] and RM Anovas (see **Results S1**).

The overall simplicity and robustness of this method being attractive, we needed assess its power and reliability, especially in actual usage conditions. The rest of this article formally establishes the validity and generality of the method.

### Organization of the Paper

We describe seven series of Monte-Carlo studies that assess the power, reproducibility and robustness of the UKS test procedure with individual one-way Anovas and Kruskal-Wallis tests, and compare them with the outcomes of RM Anovas or multilevel mixed-effects analyses applied to the same synthetic datasets. In these simulations, unless stated otherwise, we systematically varied the number of individuals, the number of factor levels, the number of repetitions per level, the trial-to-trial variance (for a given factor and individual), and the across-individual variance of the effect. The factor effect was zero for all individuals in simulations aimed at assessing type I errors, and non-zero in at least some individuals (several scenarios were simulated) when assessing type II errors. Part 1 and 2 evaluate the type II error rates when individual effects have Gaussian (Part 1) or mixed Gaussian distributions (Part 2). We then show that as a distribution test, the UKS test is less sensitive to exceptional individuals than alternative tests (Part 3). Next, we examine type I error rates when individual tests assumption holds (Part 4), when the homoscedasticity assumption is violated (Part 5), and when individual data is skewed or includes outlier trials (Part 6). We also show that the UKS test can be used in conjunction with non-parametric individual tests (Part 7). We finally determine the designs for which the UKS test is more appropriate than multilevel mixed-effects analyses (Part 8). Altogether, these studies provide practical guidance as to 1) the situations where UKS test procedure is better suited than RM Anova and multilevel mixed-effects analyses, 2) the optimal experimental designs for the UKS procedure, and 3) the violations of assumptions that may increase type I errors.

## Results

### 1. Power as a Function of Inter- and Intra-individual Variances

This section and the following one investigate the power of the UKS test procedure with Monte-Carlo studies. In this part, we considered the usual hypothesis that individual differences in factor effect have a Gaussian distribution: this happens when these differences result from multiple small variations. As a reference for judging power, we provide the type II error rates of RM Anovas for the same datasets. Note that both procedures are not equivalent, as stressed above. Although UKS and Anovas apply to the same doubly repeated measure experimental designs and both test the effect of experimental factors on the variable of interest, the UKS test assesses the *global null hypothesis* while RM Anovas assesses the *null average hypothesis* to evidence main effects. Comparing the two methods can help choosing between hypotheses from preliminary or similar experiments, and optimizing the experimental design for either RM Anova or UKS test.

**Figure 1:**
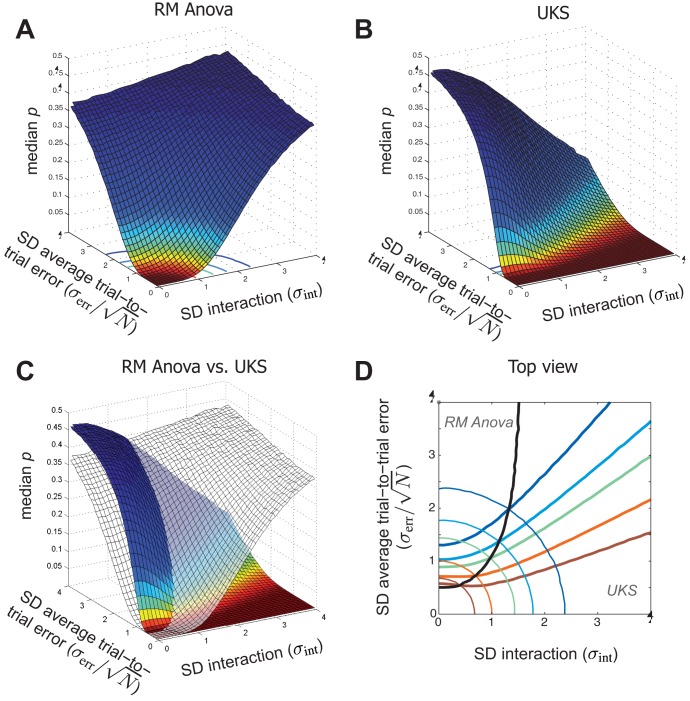
Comparison of type II error rates in UKS test and RM Anovas . Results of a simulation study based on over one billion datasets. Each dataset represents the data of 10 individuals performing 10 trials in each of the 2 levels of a factor. Each data point was obtained by adding to the fixed central value of the level (−1/√2 or +1/√2) two random Gaussian values representing individual idiosyncrasies and trial-to-trial errors (see Methods). *Panel A:* Median probability (Z-axis) yielded by RM Anovas as a function of the standard deviations of subject-factor interaction (X-axis, rightwards) and average of 10 trial-to-trial errors (Y-axis, leftwards). *Panel B:* Median probability yielded by the UKS test for the same random data. *Panel C:* superimposition of the surfaces displayed in panel A and B. Note that in conditions when UKS test is less powerful than ANOVA (larger median p), the difference in power is never dramatic; the converse is not true. *Panel D:* 2D-isolines of the surfaces in panel C for median probabilities. 001 (red), .01 (orange), .05 (green), .10 (light blue) and .20 (dark blue). Black line: projection of the intersection of the two surfaces; RM Anova is more powerful (smaller median probability) than the UKS test for points leftwards of the black line. Note that scaling the X-axis to the SD of within-level averages of trial-to-trial errors gives a symmetrical aspect to RM Anova surface and projection.

In this study, we first assessed how the median probabilities yielded by UKS tests and RM Anovas varied as a function of intra- and inter-individual variability for a ‘typical’ design (a single two-level fixed factor, 10 individuals and 10 trial repetitions). Random datasets were obtained by adding three values: a constant effect (−1/√2 in level 1, +1/√2 in level 2); a random individual effect drawn for each level from a Gaussian distribution with null mean and variance σ_int_^2^; and a random trial error drawn from a Gaussian distribution with null mean and variance σ_err_^2^. We computed the median probability yielded by 100000 UKS tests and RM Anovas for every combination of 41 values for σ_int_, the standard deviation of the interaction between individuals and fixed factor, and 46 values for σ_err_, the standard deviation of trial-to-trial errors (see **Methods** for details). The results are displayed in Figure 1, with axes units chosen to give a cylindrical symmetry to the median probability of RM Anovas. The scales of the horizontal axes correspond to a unitary effect size *S*_eff_. The shapes are invariant if *S*_eff_ and the scale of the two horizontal axes are multiplied by a common factor. This enables results from any preliminary study to be situated in Figure 1 after dividing estimates of σ_int_^2^ and σ_err_^2^ by an estimate of *S*_eff_ (see Methods). Red to green surfaces and lines denote median probabilities between 0 and 0.05, i.e. type II error rates below 0.50 at the 0.05 threshold.

The most salient feature of Figure 1 is the difference in extension between the red-to-green surfaces of RM Anovas (panel A, circular colored lines in panel D) and the UKS test procedure (panel B, colored surface in panel C, thick colored lines in panel D). It highlights the limits of testing the *null average hypothesis* to evidence stereotypical effects. For RM Anovas, the expected values of the F numerator and denominator are respectively equal to 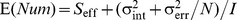 and 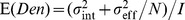 where *N* is the number of within-level repetitions and *I* the number of individuals. A rough computation shows that RM Anovas have type II error rates above 50% at the.05 threshold as soon as σ_int_^2^ exceeds 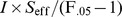 where F_.05_ is the 0.05 threshold of the F. In the present design with 2 levels and 10 individuals, this limit is reached when the standard deviation σ_int_ is approximately equal to the average difference between levels (√2). Except with high population size, RM Anovas are powerless for evidencing factors whose individual effect distribution has second moment σ_int_^2^ two or three times larger than the first moment defined as *S*_eff_.

By contrast, the UKS test procedure is suited to evidence factors that show up through the inter-individual variance of their effects σ_int_^2^. Its outcome essentially depends on the ratio of σ_int_ to σ_err_/√*N*. This is shown by the convergence of the rightward part of the thick colored curves in Panel D towards a point close to the origin. These curves indicate constant median probability for the UKS procedure. Their asymptotes for large σ_int_ are lines that converge towards the point of coordinate (-*S*_eff_/2, 0). This geometrical feature would be observed for any experimental design (see Supporting Information for details). Therefore, it demonstrates that the UKS procedure can yield low type II error rate with moderate number of within-level repetitions as soon as σ_err_ is small enough compared to σ_int_.

The left part of Panel C in Figure 1 enables to compare the type II error rates of the two procedures when there is not any inter-individual variability (σ_int_ = 0). In these situations, the *global null hypothesis* and the *null average hypothesis* are equivalent, i.e. both of them are either true or false in the population. When the level of trial-to-trial variability is large (relatively to effect size), the RM Anovas’ advantage of averaging individual effects and pooling errors is clearly visible as a median probability lower than that yielded by the UKS test. However, as σ_err_/√*N* decreases, the advantage decreases and eventually disappears. Thus, the UKS test procedure has higher type II errors rates than RM Anovas when both tests have little chance to evidence factor effects. When the median *p*-value of RM Anovas is equal or smaller than.05, the range of (σ_err_, σ_int_) duplets where the UKS test has higher median *p*-value shrinks as σ_err_ decreases (areas between thin and thick homologous lines in Panel D). Eventually, when σ_err_/√*N* is low with respect to effect size, the UKS test procedure have better sensitivity than RM Anovas (Panel C) if σ_err_ varies across individuals, as in the present simulation (see **Methods**) and in most experiments. The advantage of pooling residual errors may turn to a disadvantage when residual errors arise from a mixed Gaussian distribution [15].

To explore the effect of the experimental design, we finally carried out 40 additional simulations varying the numbers of individuals (4 to 40), factor levels (2 to 5) and intra-level repetitions (2 to 100). We first found that increasing the number of individuals beyond 8 benefits similarly to the UKS test and RM Anovas, while decreasing it below 8 increases more the type II error rates in RM Anovas than in the UKS test. A second – more expected – finding was that increasing the number of trials by individual (numbers of levels multiplied by the number of within-level repetitions) decreased more the type II error rate for the UKS test than for RM Anovas. We conclude that when high inter-individual variability of the effect suggests using the UKS test procedure, it is sensible to plan a large number of trials by individual rather than a large cohort of individuals, if the total number of trials is a constraint.

As a general conclusion to this Part 1, our Monte-Carlo analyses show that the UKS method is largely as powerful for testing the *global null hypothesis* as is the standard RM Anova for the *null average hypothesis*.

### 2. Power in a Heterogeneous Population

In the above simulation studies, we assumed that individual effects had Gaussian distribution. However, in many situations mixed Gaussian distributions are more plausible. For example, the behavior of individual subjects may depend on their gender or cultural origin; the investigated system may have two or more equilibrium states or local minima; the experiment may be carried out by several experimenters; morning experimental sessions may provide different results than afternoon ones because of differences in room temperature or subject’s arousal. The principle of mixed Gaussian distributions of individual effects encompasses all these situations and many others. In such circumstances, one can wonder at which point inferences drawn from RM Anovas and UKS test are reproducible and generalizable. To get insight into this issue, we carried out simulations to assess type II error rates for a design with 10 individuals, a 2-level factor and 8 within-level repetitions. In these simulations, we modeled population heterogeneity as the mixture of two subpopulations of subjects, a subpopulation showing an effect *d* of the factor, and one showing no effect. A third subpopulation of subjects showing on average an opposite effect was occasionally added. Thus, in our Monte-Carlo simulations the trial-to-trial variability was constant while two parameters varied: the effect size, defined as the difference *d* between the two factor levels, and the proportions of population that displayed the average effect *d*, no effect, or occasionally an average opposite effect –*d* (see **Methods** for details).

**Figure 2:**
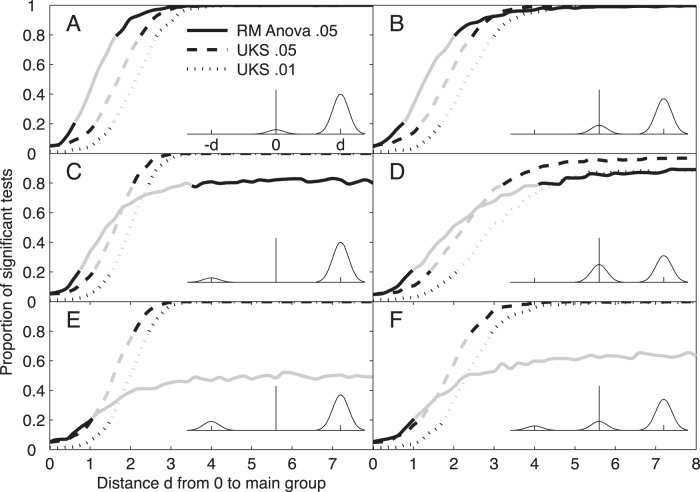
Type II errors and reproducibility with heterogeneous experimental effects . Each panel displays the proportion of significant hypothetical experiments as a function of the difference *d* between the constant values of experimental effect in 2 (panels A–E) or 3 sub-populations (panel F). The lines show the proportion of significant tests in 10000 hypothetical experiments for 41 values of *d* from 0 to 8 by .2 steps for RM Anovas (continuous line) and the UKS test at both the .05 (dashed line) and.01 threshold (dotted line). The gray part of lines indicates the 0.211–0.789 range of proportion of significant tests for which the probability that two subsequent experiments yield conflicting outcomes exceeds 1/3. Each experiment consists in 10 individuals performing 8 trials in a baseline condition and in an experimental condition. Trial errors are drawn from a Gaussian distribution with parameters 0 and √8, so that the average of the experimental condition has a Gaussian distribution centered on –*d*, 0 or +*d* (Insets) with unitary variance. The proportion and center of the subpopulations varied across studies. In the first study (*panel A*), the experimental effect was set to 0 for 10% of the population, and to *d* for the remaining 90%. In the other studies (*Panels B–F*), the effects and proportions were as follows: [0, 20%; *d*, 80%]; –*d*, 10%; *d*, 90%]; [0, 40%; *d*, 60%]; –*d*, 20%; *d*, 80%]; [–*d*, 10%; 0; 20%; *d*, 70%]. For each hypothetical experiment, the 10 individual effects were drawn with replacement from a set of –*d*, 0 and +*d* values in the above proportions (for *d* = 0, the proportion of significant tests is equal to the nominal type I error rate). We conclude that when factor effects vary across individuals as modeled by a mixture of Gaussians, UKS tests yield more reproducible outcomes than RM Anovas and have lower type II errors.

Panels A and B in Figure 2 show the proportion of significant RM Anovas (continuous line) and UKS tests at the.05 (dashed) and.01 thresholds (dotted) for two simulation studies where the experimental effect was null in 10 and 20% of the population, respectively, and equal to *d* in the rest of the population. As the value of the effect size *d* in the bulk of the population increased from 0 to 8, all three lines increased from the nominal type I error rate to the value 1 associated with null type II error rate and perfect reproducibility. The horizontal shift between curves reflects decreasing power from RM Anova to UKS test at the.01 threshold (the power difference would be smaller if non-null individual effects were variable rather than all equal to *d*). Grey lines indicate low reproducibility defined as probability beyond 1/3 that two independent experiments yield conflicting outcomes. If *p* is the proportion of significant outcome, low reproducibility occur when *p* ^2^ + (1−*p*)^2^ >1/3, i.e. for *p* between 0.211 and 0.789. In panel A and B, all three tests have low reproducibility (grey line) for a similar span of experimental effect values. In panel C (10% of the population with effect equal to –*d*) and D (40% with 0), RM Anovas has reproducibility below 2/3 (grey zone) for a larger range of effect values than UKS test. In addition, for whatever large effect in 90% (C) or 60% (D) of the population, the type II error rates cannot decrease beyond 20% (C) or 10% (D) for RM Anovas, while the minimum is 0% (C) or smaller than 10% (D) for the UKS test. The reproducibility advantage of the UKS test is even higher when 20% of the population display a –*d* effect and 80% a +*d* effect (E), as well as when 10% display a –*d* effect, 20% a 0 effect and 70% a +*d* effect (F). As a whole, these simulations studies demonstrate that in situations where individual effects are have mixed Gaussian distributions, the UKS test yields more reproducible outcomes than RM Anovas and has lower type II errors when the effect size is large enough.

These simulations also provide an insight into the proportion of individuals in the population that show a significant effect when UKS tests are significant at the .01 or .05 level. In panel D (60% of significant effects), it can be seen that type II errors never disappear as *d* increases. Other simulations (not shown) indicate that for this specific design the probability for the UKS tests to be significant cannot exceed 50% when the effect is null in 60% or 72% of the population when the .01 and .05 threshold are used, respectively. This is not unexpected for a test designed to assess differences between distributions. Moreover, the probability for the UKS test to be significant cannot exceed 5% for the 0.01 threshold (16% for 0.05) when the effect is null in more than 88% of the population. This shows that the UKS procedure is fairly resistant to outlying individuals. We will develop this point in Part 3.

We carried out additional Monte-Carlo studies to determine the population size and UKS threshold level for which it could be stated that “at least 10% of individuals show an effect” with less than 5% chance of being wrong. For less than 22 individuals, the statement holds if the UKS test yields a *p*-value smaller or equal to .01. For population size between 23 and 43 individuals, the UKS test must yield a *p*-value smaller or equal to .005. These 10% statements do not depend on the experimental design except for population size, because they rely on the *p*-values of individual tests but not on the nature of the tests. In addition, our estimations were obtained using high values for the experimental effect *d* (8 times the s.d. of levels’ average). With smaller experimental effects, it would need more than 10% of the population to make the UKS test significant at the .01 threshold with less than 5% chance of being wrong. To summarize, when the UKS test rejects the *global null hypothesis*, beyond the formal conclusion that there is at least one non-null individual effect, it seems legitimate to infer that individual effects are not null in a non-negligible proportion of the population.

### 3. Robustness of UKS Test with Respect to Outlying Individuals

Robustness with respect to outlier individuals, namely resistance, is the first quality required for a statistical test intended to support population inference. Indeed, one would not trust a test that yields false positives by rejecting the null hypothesis when there is a large effect in only one or two individuals. Symmetrically, a trustful test should reject the null hypothesis when there is a large effect in all individuals except one or two. In this Part, we investigate the impact of outlying individuals, i.e. exceptional individuals for which the effect of the investigated factor is genuinely different from the effect in the population. We show here that the UKS have the required robustness against this source of type I and type II errors. We also establish that this robustness is lacking to all of the numerous methods for combining *p*-values proposed to date for meta-analytic studies or for the same purpose as the UKS test.

The one-sample Kolmogorov-Smirnov test assesses whether a sample is likely to be drawn from a theoretical distribution. It is based on the largest difference between the empirical and theoretical cumulative distributions. We use it to assess whether *p*-values are uniformly distributed between 0 and 1: the unilateral test allows rejecting the hypothesis that there is no individual effect by showing that the distribution of individual *p*-values is abnormally biased towards small *p*-values. The UKS test statistic is 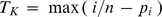 where *p_i_* is the i^th^ *p*-value in increasing order and *n* the population size. The UKS test is resistant because *T_K_* cannot reach the .05 threshold unless at least three or more *p*-values are below a low limit that varies with population size. For example, with a sample of 10 *p*-values, the UKS test is not significant at the .05 threshold (T_K_ <0.369) unless there are 4 individual *p*-values below .031 (4/10 – 0.369). Based on the Kolmogorov distribution, we computed for different population sizes the minimum number of *p*-values necessary for the set to be significant at the .01 and .05 threshold (Table 1). This minimum number of *p*-values asymptotically tends towards 1.224×√*N* for the .05 threshold, and towards 1.517×√*N* for the .01 threshold [16]. The formula for computing *T_K_* makes the test robust also with respect to type II errors: it is clear that one, two or three high outlier *p*-values cannot prevent the UKS test to yield a significant outcome if most individual tests result in low *p*-values. This two-sided robustness of the UKS test with respect to outlier *p*-values is unique among numerous alternative methods for combining independent *p*-values.

**Table 1:** UKS test thresholds and associated p-value limits.

Pop. size	.05 threshold			.01 threshold		
	T_K_ thresh.	Min nb	p-value	T_K_ thresh.	Min nb	p-value
**5**	0.50945	3	.09055	.62718	4	.17282
**6**	0.46799	3	.03201	0.57741	4	.08926
**7**	0.43607	4	.13536	0.53844	4	.03299
**8**	0.40962	4	.09038	0.50654	5	.11846
**9**	0.38746	4	.05698	0.47959	5	.07596
**10**	0.36866	4	.03134	0.45662	5	.04338
**12**	0.33815	5	.07516	0.41918	6	.08082
**15**	0.30397	5	.02936	0.37713	6	.02287
**20**	0.26473	6	.03527	0.32866	7	.02134
**30**	0.21756	7	.01577	0.27023	9	.02977

For ten population sizes *I* from 5 to 30 individuals, the table indicates the Kolmogorov-Smirnov test threshold K_th_ for type I error rates equal to .05 (column 2) and .01 (column 5). Column 3 and 6 indicate the minimum number *n*_min_ of *p*-values required for the UKS test to be significant. These *p*-values have to be lower than the limit p_min_ indicated in columns 4 and 7. Note that the UKS test is significant as soon as *n*_min_ + *m p*-values are below p_min_ + *m*/*I* for any m between 0 and *I*-*n*_min_. By construction, the limit for *I p*-values is equal to 1-K_th_.

Many methods have been proposed for combining independent *p*-values in meta-analytical studies (reviews in references [17]–[19]). The most popular ones were devised by Fisher [8] and Stouffer and colleagues [9]. These two methods deserve special attention because two independent groups have proposed them to be used in fMRI studies for the same purpose as UKS test here, i.e. as alternative to mixed-effects analyses [2], [3], [5]–[7]. The Fisher’s statistic is *T_F_* = –2×log (*P*), where *P* is the product of *n* independent *p*-values. *T_F_* follows a χ2 distribution with 2*n* degrees of freedom. It is easy to see that a single arbitrary small *p*-value can make *T_F_* arbitrary high and its probability arbitrary close to 0. The method proposed by Stouffer and colleagues [9] is based on the statistic
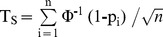
 where Ф^-1^ is the inverse normal cumulative distribution function. If the *global null hypothesis* holds, *T_S_* follows the standard normal distribution. The formula for computing *T_S_* shows the high sensibility of Stouffer’s to for both small and high outlier *p*-values. On the one hand, a sufficiently low p-value can make *T_S_* significant even if the other *p*-values have uniform distribution between 0 and 1. On the other hand, a *p*-value arbitrary close to 1 can pull down *T_S_* below the significant threshold even if all other *p*-values are close to 0. Almost all other methods for combining *p*-values are similarly sensitive. A single individual outlier may cause type II error in methods based on the sum of the *p*-values [20], the maximal p-value [10] and the product of the *p*-values minus one [21], and type I error in a method based on the minimal *p*-value [10]. Only a method based on the number of *p*-values below the .05 threshold [22] is robust with respect to both types of error. However, the fixed .05 threshold of this method similar to the UKS test makes it clearly less appropriate for the goal of evidencing individually variable effects. Overall, the UKS test is probably the most robust method to combine the results of individual tests.

### 4. Reliability with Equal within-level Variances and Gaussian Data

Researchers who are not professional statisticians may wonder whether it is safe to make statistics on statistics. More specifically, although the reliability of both independent-measures one-way Anovas and KS tests are beyond any doubt if their respective assumptions are met, it may be asked whether chaining them yields a normal rate of rejection of the null hypothesis. From a theoretical viewpoint, this is not an issue. If the *global null hypothesis* holds and Anovas’ assumption are fully met, then individual *p*-values will be uniformly distributed between 0 and 1, and the UKS test at the .05 threshold will yield 5% of false positive To illustrate this point, we begin with a Monte-Carlo study of type I error rates when assumptions for all tests are met. Specifically, we estimated the type I error rate of the UKS test procedure for 168 one-way Anova designs with different numbers of individuals, factor levels and repetitions, and with trial-to-trial errors drawn from a single Gaussian distribution. In this and other type I errors rate studies involving comparison with RM Anovas, both the effect and its variability across individuals σ_int_ were set to zero (see **Methods** for details).

As expected, for the nominal 0.05 threshold, we found that UKS test wrongly rejected the null hypothesis for 4.9931% of the random sets, while the rejection rate was 4.6346% for the RM Anova (the smaller rejection rate for the RM Anova reflected loss of power due to inter-individual variations of σ_err_, the standard deviation of trial-to-trial errors [15]; running the same Monte-Carlo analyses with the same standard deviation for all individuals yielded average type I error rates of 4.9981 and 4.9893% for the RM Anovas and the UKS test procedure, respectively). For a .01 threshold, the average type I error rates amounted to 0.9965% and 0.8503% for UKS test procedure and RM Anova, respectively. The behavior of the UKS test conforms to what is expected when the assumptions of individual tests are met. The question now arises how the UKS test behaves when these assumptions are violated.

### 5. Reliability in the Presence of Violations of Homoscedasticity in Individual Anovas

**Figure 3:**
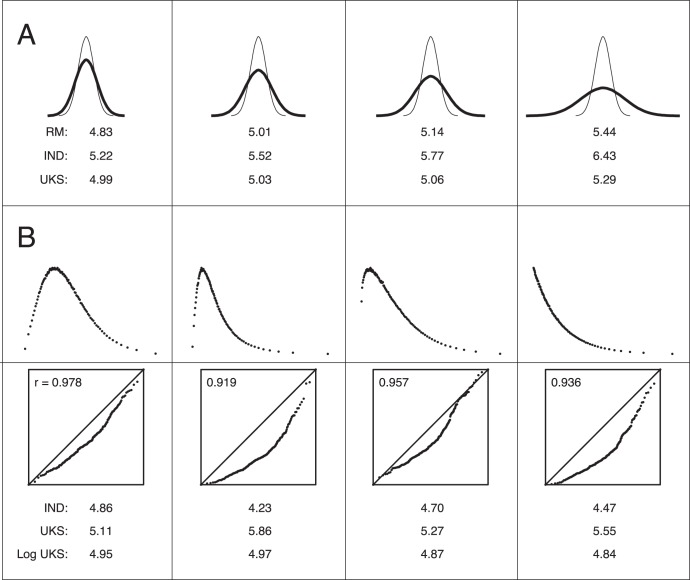
Violations of homoscedasticity and normality assumptions in one-way Anova design: compared robustness of RM Anova and UKS test. *Panel A:* Violation of equal variance assumption. Curves display trial-to-trial errors distributions in the factor levels with the smallest and largest variance for the 4 degrees of heteroscedasticity investigated in simulation studies (see Methods). The numbers under the curves indicate the average percentage of type I errors (false positives) for RM Anovas, individual Anovas and the UKS test procedure, respectively. Numbers above 5% indicate an excess of significant datasets with respect to the tests threshold (0.05). We observe that the UKS test, as the RM Anova, is robust to heterogeneity of variance. *Panel B:* Violation of normality assumption. Curves display the empirical distributions of trial-to-trial errors drawn from the following 4 distributions: gamma with k = 4; lognormal with μ = 0 and σ = 1/√2; Weibull with k = 1.2 and λ = 0.5; exponential with λ = 0.4 (see Methods). Boxes: Normal probability plots of typical residuals from an Anova applied to skewed data randomly drawn from the above distribution. For the displayed residuals (10 individuals × 3 levels × 10 repetitions with a median coefficient of correlation r), skewness is significant at the .01 threshold when r <0.9942. The numbers under the boxes indicate the across-designs average percentage of type I errors (false positives) for individual Anovas and UKS test applied to raw data or after a logarithmic transformation. Numbers above 5% indicate an excess of significant datasets with respect to the threshold used (0.05). When data is skewed, the UKS test should be used in conjunction with individual nonparametric tests (see text, Part 7), or data should be (log-)transformed.

Independent-measures (IM) Anovas rely on the assumption that residual errors have Gaussian distribution with equal variance across factor levels, but they are robust with respect to non-normality and to moderate heteroscedasticity [23]. However, it is not clear whether and how much violation of either assumption can affect the outcome of the KS test applied to the probabilities yielded by individual Anovas. In this section, we focus on violations of the homoscedasticity assumption. When the factor effect is null, the probabilities of individual Anovas with unequal variances are not uniformly distributed on the [0 1] interval. Rather, they have more than 5% chance to be smaller than 0.05. This systematic bias might be enhanced because the Kolmogorov-Smirnov tests assess whether individual probabilities are significantly smaller than they should if drawn from uniform distribution on [0 1]. To investigate this issue, we computed the type I error rates of UKS test, IM and RM Anovas for 216 different random datasets to assess how these rates varied as a function of the level of heteroscedasticity and of the numbers of individuals, fixed-factor levels and within-level repetitions. The levels of heteroscedasticity were defined by the ratio of the largest to the smallest variance (2, 3, 4 or 8). The results confirmed that in individual IM Anovas the rate of false positives is abnormally high and increases as the heteroscedasticity increases (Figure 3, panel A, line IND). However, this bias, far from being enhanced, was watered down by the UKS test (see Supporting Information for further explanation).

On average, both UKS test procedure and RM Anova proved robust with respect to violation of the assumption that trial-to-trial variability was constant across factor levels (Figure 3, panel A). However, the reliability of the two procedures appeared to be slightly affected in specific and different contexts (Table 2). In line with the biased distribution of individual Anova probabilities, the UKS test was more sensitive to heteroscedasticity when there were only few trials per individual. The rate of false positives was abnormally high when there were less than 10 repetitions in 2-level factors (example in Table 2) or less than 3 repetitions in 3-level factors. The excess of type I errors increased as the number of individuals increased from 5 to 20. Additional analyses show that assessing heteroscedasticity with Levene’s or Bartlett’s tests was of little help to prevent this risk (Supporting Information). In contrast with the UKS test, RM Anovas was perfectly reliable for designs with 2-level factor, but was less robust with 3-level factor, and clearly sensitive to heteroscedasticity with 4-level factor (example in Table 2). This excess of false positives was due to violations of the sphericity assumption: unequal trial-to-trial within-level variances resulted in unequal inter-individual variances of level-averaged data.

**Table 2:** Robustness with violations of heteroscedasticity assumption.

Designs	Ratio of the largest to the smallest variance			
	**2**	**3**	**4**	**8**
**3 trials × 2 levels:**				
RM Anovas	4.67	4.72	4.74	4.72
UKS test	5.32	**5.76**	**6.11**	**7.37**
**10 trials × 3 levels:**				
RM Anovas	4.90	4.94	5.20	**5.50**
UKS test	4.78	4.65	4.43	4.17
**5 trials × 4 levels:**				
RM Anovas	4.98	5.28	**5.54**	**6.13**
UKS test	4.79	4.81	4.69	4.64

Rates of type I errors in repeated-measures Anovas and UKS test for 3 representative experimental designs (lines) and the same 4 degrees of heteroscedasticity as in Figure 3A (columns). Rates are averages of designs with 5, 10 and 20 individuals. The rates of each design are equal to the percentages of 60000 random datasets found significant at the 0.05 threshold as the effect of factor was set to zero. Bold values indicate large excess of type I errors. UKS (and RM Anova) are globally robust to violations of heteroscedasticity.

### 6. Reliability in the Presence of Violations of Normality in Individual One-way Anovas

Skewness and outlier trials in individual Anovas can affect the UKS test type I error rate as heteroscedasticity. In the Monte-Carlo simulations of this section, we systematically investigated non-normality with 13 types of non-Gaussian distributions of individual data (Gaussian distributions were also used as a baseline). Non-Gaussian distributions included 8 skewed distributions (gamma, lognormal, Weibull and exponential distributions, each with two different set of parameters), as well as 5 Gaussian distributions with different proportions and levels of outliers. These samples were simulated in 84 one-way Anova designs to encompass most practical situations (designs were characterized by 5 to 20 individuals, 2 to 4 factor levels and 2 to 32 within-level repetitions). For every couple of distribution and design, we computed the type I error rates of UKS test, IM and RM Anovas. We compared them with nominal rates and with the rates computed for 3 baseline Gaussian distributions. We found that the UKS test had excessive type I error rates for a large range of designs. Nevertheless, the type I error rates was brought back to nominal level by prior logarithmic transformation of individual data.

We first report results for skewness. RM Anova revealed perfectly robust, with rates of type I errors that never exceeded 5.1%. By contrast, we found that the UKS test was *not* robust for skewed data (see Supporting Information for explanations). More specifically, type I error rates were particularly abnormal when the number of individuals was high (e.g. 20), the number of factor levels low (e.g. 2), and the number of repetitions in the 5–10 range. As concerns distributions, type I error rates increased as the coefficient of correlation of the normal probability plot decreased (insets in Figure 3). Table 3 displays the rate of false positives for 3 representative experimental designs with the distributions displayed in Figure 3B. With 5 individuals, 4 factor levels and 32 repetitions, the UKS test was quite robust against positive skewness. However, for the distribution with the smallest coefficient of correlation of the normal probability plot (lognormal distribution, column 2), the type I error rates already exceeded the across-design maximal rate observed with the 3 baseline Gaussian distributions (5.19%). With 10 individuals and 3 factor levels, the error rates exceeded this maximal value even for the least skewed distribution (gamma, column 1). In the worst case that we found (20 individuals, 2 factor levels, 5 trials), the rate of type I errors was strongly biased for all 4 distributions shown in Figure 3B and for the 4 other distributions covered by our simulation study. We conclude that the UKS test should not be applied to IM Anovas of skewed individual data except in designs similar to the line 1 in Table 3. When skewness is suspected, one of the two following methods can be safely applied. First, and simplest, the individual Anovas can be carried out after a logarithmic transformation of the data. After such a transformation, for all skewed distributions and all designs we tested, the rate of false negative dropped to the nominal values of the .01 and .05 thresholds (see Table 3 and bottom values in Figure 3B). Second, and most powerful when the data is strongly skewed and when there are at least 15 or 20 trials per individual, the UKS test can be applied with individual Kruskal-Wallis tests instead of one-way Anovas (see below).

**Table 3:** Robustness with skewed data.

Distributions:	1	2	3	4
**5 subj. × 4 levels × 32 trials:**				
UKS test	5.00	5.34	5.03	5.01
Log transformation	5.07	5.07	5.01	4.91
**10 subj. × 3 levels × 10 trials:**				
UKS test	5.36	**6.17**	**5.27**	**6.09**
Log transformation	5.01	4.93	4.72	5.10
**20 subj. × 2 levels × 5 trials:**				
UKS test	5.60	**8.61**	**6.62**	**7.85**
Log transformation	4.98	5.22	4.68	4.66
UKS - Kruskal-Wallis	2.12	2.11	2.08	2.11

Rates of type I errors in UKS test for 3 representative experimental designs (lines) and the 4 skewed distributions shown in Figure 3B (columns). In each design, the UKS test was applied before and after log-transforming the random datasets. The rates of each design are equal to the percentages of 60000 random datasets with null factor effect that were found significant at the 0.05 threshold by the UKS test. The type I error rates obtained for the same data with Kruskal-Wallis test substituted to Anova are also indicated for the third design. Overall, either log-transformation of skewed data or use of a per-individual nonparametric test guards the UKS test against excessive type I errors.

Regarding the effect of outliers on the reliability of the UKS test with one-way Anovas, we found that 2.5% or 7.5% of indiscernible outliers between +2 and +3 standard deviations from the mean did not increase the rate of type I errors. The same proportions of unilateral removable outliers (between +3 and +4 s.d.) increased the rate up to 6.6%, and 2.5% of removable outliers on both sides of the distribution also resulted in a small excess of false positives. However, the excess of type I errors was negligible for designs with 15 or 32 within-level repetitions, or with 4 levels and less than 10 individuals. We conclude that it is safe to systematically remove outliers beyond 3 standard deviations before applying the UKS test to individual Anovas.

### 7. UKS Test with Individual Kruskal-Wallis Tests

Applying the UKS test to the *p*-values yielded by non-parametric tests is an appropriate solution when individual data violate the assumptions of parametric tests. In this respect, three points deserve attention. First, non-parametric tests are not fully free of assumptions. For example, the Kruskal-Wallis test requires that the investigated variable have an underlying continuous distribution. Second, statistical packages often provide approximate statistics that do not suit the UKS test. The possibility to compare the Kruskal-Wallis statistic to the critical values of a chi-square distribution when there are more than 5 trials in each condition level [24] does not imply that the distribution of the Kruskal-Wallis statistic is identical to that of a chi-square with the appropriate number of degrees of freedom. To corroborate this point, we computed the type I errors with approximate and exact *p*-values for a large number of random datasets with 7 trials in each of 3 factor levels. We found that the rate of type I errors at the .05 threshold amounted to 6.2 or 4.9% depending on whether the probability of the individual Kruskal-Wallis statistic was derived from the chi-square approximation or from the exact distribution. As a general rule, we recommend to use exact distributions, or good Monte-Carlo approximations of them, when applying the UKS to the *p*-values of individual non-parametric tests. Third, the power of a non-parametric test can be higher than that of a parametric test.

To illustrate the latter point, we estimated both type I and type II error rates of the UKS test with individual Kruskal-Wallis tests for a variety of skewed distributions and single-factor experimental designs. As expected, we found that the procedure was fully reliable, with rates of type I error that never exceeded 5.2% across the 384 tested combinations of designs and skewed distributions. Table 3 indicates the rates obtained for the 20×2×5 design with the skewed distributions shown in Figure 3B. In addition, we compared the type II error rates of the UKS test when the same individual datasets were assessed with a Kruskal-Wallis test or with Anovas before and after logarithmic transformations. With Gaussian data, as expected from the loss of information between interval and ordinal measures, the procedure with the Kruskal-Wallis test was always less powerful than the 2 others, specially for designs with few repetitions and levels. However, with skewed data, the procedure with the Kruskal-Wallis test was the most powerful as soon as the number of repetitions exceeded 4 or 5 (4- and 3-level designs) or 9 (2-level). It remained less powerful than with Anovas for designs with few repetitions and levels, especially for the 2×2, 3×2, and 2×3 designs. We conclude that if individual data are skewed, applying the UKS test to individual Kruskal-Wallis tests is the best way for assessing the *global null hypothesis*, provided that the experimental design includes at least 15 trials (in total) per individual.

### 8. Choosing UKS or Multilevel Mixed-effects Analyses According to Sample Sizes

In addition to RM Anovas and UKS test, repeated-measures designs datasets can also be analyzed using multilevel mixed-effects (ME) models. However, it is unknown whether the latter procedure is suited for designs with small number of individuals or repetitions. Indeed, while ME analyses have been shown to require at least 30 to 50 individuals for yielding accurate estimates in regressions [11]–[14], we are not aware of similar investigations for RM Anovas designs. Therefore, we used Monte-Carlo simulations to compare the type I and type II error rates in ME analyses and UKS tests. These investigations lead us to the conclusion that the UKS test should be preferred to ME analyses in studies that include less than 30 to 50 individuals.

From the viewpoint of ME analyses, RM-Anova designs involve datasets with three hierarchical levels and as many random variables: trials are nested in experimental conditions that are themselves nested in individuals. For example, in educational studies (where the UKS test can also be an alternative to multilevel ME analyses), pupils can be nested in types of classes themselves nested in different schools. Keeping the same notations as in the other sections, ME analyses rely on the following assumptions. At the lowest hierarchical level, errors have the same Gaussian distribution with null average and variance σ_err_^2^ across all individuals and conditions. At the middle hierarchical level, the individual effect of the j^th^ experimental condition follows a Gaussian distribution with mean µ_j_ and condition-independent variance σ_int_^2^. At the highest hierarchical level, individual average follows a Gaussian distribution with parameters µ_subj_ and σ_subj_^2^. The gist of ME analyses is to estimate these parameters and their confidence intervals (CI) by means of an iterative convergence process that maximizes their likelihood. When the goal is to assess whether the experimental factor affects individual behaviors, ME analyses involve deciding between a full and a restricted model which assumes that σ_int_ is null, i.e. that trial-to-trial errors are the only source of inter-individual differences in experimental condition averages. The restricted model is assessed when the full model, always tested first, does not reject the H_0_ hypothesis that σ_int_ is null. The restricted model tests the across-individual average of the factor’s effect against the trial-to-trial errors; this amounts to pooling together the data of all individuals after having subtracted the individual across-condition averages. As a result, the restricted model is potentially more powerful than a RM Anova because its F statistic has the same numerator but more degrees of freedom associated with the denominator. For example, in a 10 subjects × 2 levels × 10 repetitions design, a RM Anova uses a F(1,9) test while the restricted model uses a F(1,189) test. This test is legitimate if the variations in individual factor’s effect result exclusively from trial-to-trial errors. In the converse case, the test of the restricted model will inflate type I error rates above the nominal threshold because the F distribution has more degrees of freedom than it should. To summarize, type II errors in testing the full model are likely to lead to excessive type I error rates in the restricted model. The present Monte-Carlo study aimed to assess the type II error rates (power) in testing the full model, as well as their consequences for type I error rates in the restricted model. This was done in 490 random datasets of varying number of individuals, factor levels, within-level repetitions, and partial Intraclass Correlation Coefficients (pICC). We explain below the rationale for systematically varying this coefficient, defined as pICC = σ_int_^2^/(σ_int_^2^+ σ_err_^2^/*N*) where *N* is the number of within-level repetitions [23]. The effect of the factor was randomly drawn from Gaussian distributions with null average and null or non-null variance (thus pICC). For each design, we assessed the type II error rates of the full model and their causes (proportion of unavailable and ill-defined confidence intervals), the significance and type I error rates in the restricted model, and the type I and II error rates of the UKS test for the same datasets.

In preliminary simulations, we systematically varied the classical intraclass correlation coefficient ICC, defined as σ_int_^2^/(σ_int_^2^+ σ_err_^2^), because it does not depend on the design. We switched to investigate the effect of pICC because the ratio of σ_int_^2^ to σ_err_^2^/*N* largely determines, together with the number of individuals, the type I and II error rates. To grasp this point, recall that the expected value of the empirical variance of levels’ averages is equal to σ_int_^2^+σ_err_^2^/*N*. Therefore, the estimation of σ_int_^2^ relies on the difference between the empirical variance of level averages and the empirical variance of trial-to-trial errors divided by the number of repetitions *N*. When σ_int_^2^ is smaller or hardly larger than σ_err_^2^/*N*, and the numbers of individuals and repetitions small, the above difference can happen to be close to zero or even negative. In such situations the iterative estimation procedure either cannot converge or yields ill-defined confidence intervals for the variance ([14], [25], [26], see also Tables S1 and S2). Therefore, a comprehensive analysis of type I and II error rates in ME analyses required testing the specific influence of the ratio *R* = σ_int_^2^/(σ_err_^2^/*N*), or, equivalently, of the pICC value.

We now report the result of the simulation studies. First, we found that the values and CI of all μ and σ parameters *but* σ_int_^2^ were generally accurately estimated in our fully balanced random datasets drawn according to the assumptions of the mixed-effect models (see Supporting Information for details). The only problem concerned the estimation of the effect variance CI and consequently the power for evidencing non-null σ_int_^2^ when the pICC was low or when the number of individuals was small. In these situations, the estimation of the CI frequently failed altogether or was abnormally large (see Supporting Information). This resulted in a low power of ME analyses for evidencing significant random effect components when the pICC was small, i.e. when the across-individuals effect variance σ_int_^2^ was low with respect to the error variance σ_err_^2^ and the number of repetitions inadequately small (see Table S3). More precisely, the power was below 10% for low pICC and small number of individuals. Adequate power (80%) typically required at least 50 (2-level factor) or 15 (4-level factor) individuals, and a number of trials by level sufficient for the pICC to reach 0.5 (e.g. 3, 5, 10 and 20 trials for ICC equal to 0.25, 0.17, 0.09 and 0.05, respectively).

This lack of power can be detrimental when the missed components are large enough to bias the ensuing statistical tests –that assume these components are exactly null. To properly tackle this issue, we first investigated how the type I error rates of the restricted model varied as a function of pICC and sample sizes across *all* datasets, and then focused on the datasets with type II errors in the full model. As for the first point, we found that the type I error rate of the restricted model steadily increased with the pICC and the number of factor’s levels up to 40%, and that, at variance with type II errors in the full model, it did not depend on the number of individuals (Table S4). Finally, in keeping with this observation, we found that the percentages of datasets with no significant random effect component in the full model and a wrongly significant main effect in the restricted model were well above 5% for small and medium numbers of individuals. We stress that these rates *increased* (up to 13%) with pICC values, and thus with ICC and the number of repetitions (see Table S5).

In light of these results, what should be the minimum population size to have adequate power and keep type I errors close to their nominal rate when the restricted model is assessed after the full model failed to evidence a random effect component? From a strict viewpoint, and considering that there is no *a priori* knowledge about the ICC, at least 100 individuals in a 4-level condition design, and probably 200 with a 2-level condition, would be required to have at most 5% of datasets with a significant effect and no significant random effect component (Table S5). However, the type I error rates for 30 individuals in 4-level designs and 50 individuals in 2-level designs are smaller than 7% and exceed 5% only for pICC equal to 0.25 or smaller. A pICC equal to 0.3 corresponds to ICC equal to 0.024, 0.048, 0.091 and 0.167 for numbers of trials by level equal to 40, 20, 10 and 5, respectively. Based on the idea that ICCs smaller than 0.05 seldom occur in social and educational sciences [12] and probably when individuals are the highest hierarchical level (linguistics and psychology), we consider that designs with at least 20 trials by factor level and 30 (4-level factor) or 50 (2-level factor) individuals should yield type I error rates equal or below the nominal 5% level. It should be however noted that for these population sizes the type II error rates when testing the random effect component can be as high as 50% (Table S3) and that 50 or 100 individuals are preferable.

Since ME analyses should involve at least 50 individuals and 20 trials by factor level in RM Anova designs, would the UKS test be a sensible choice in designs with smaller sample sizes? To this end, we computed the type I and II error rates of the UKS test for the same random datasets (Table S6). As expected, we found the type I error rates equal to the nominal threshold for whatever population size. When the pICC was above zero, the power increased from 6 to 100% when the pICC, number of individuals and number of factor levels increased. It should be noted that although the power does not depend on the number of trials for a given pICC, it does increase with the number of trials by level through the pICC. Finally, we computed for all datasets the difference between the significance rates of the UKS test and random effect component test in ME analyses. The comparison showed that the two tests had comparable power, with a relative advantage for the UKS test for datasets with low number of individuals or small pICC (Table S7). More precisely, the UKS test seemed preferable to ME analyses with 6, 8, 10, 15, 30, 50, and 100 individuals when the pICC is inferior to 0.6, 0.5, 0.4, 0.35, 0.25, 0.2 and 0.15, respectively. As the ICC, and thus the pICC, is often unknown, we conclude that UKS test should be preferred to ME models for assessing datasets with less than 20 repetitions per level or less than 30 individuals (50 is there are only 2 factor levels).

Finally, we wish to stress that the above results were obtained with fully balanced datasets in which the errors of all individuals were drawn from the same Gaussian distribution, individual effects from another Gaussian distribution, and individual averages from a third distribution with a particularly high variance. Although assessing the consequences of departures from these specifications would be outside the scope of the present Monte-Carlo study, it seems likely that violation of these hypotheses would favor the UKS test rather than the ME analyses for four reasons. First, we were careful setting the variance σ_subj_^2^ over 10 times σ_int_^2^ after uncovering in preliminary studies that small σ_subj_ often result into failures in estimating the confidence intervals and biases in estimating the factor’s effect variance. In other words, the power of ME analyses can be affected when σ_subj_^2^ is smaller than σ_int_^2^ divided by the number of factor’s levels in the same way as when σ_int_^2^ is smaller than σ_err_^2^/*N* (see above). Second, the UKS test provides reliable outcome whether or not the number of repetitions varies across individuals, while estimating variances and their CI in ME analyses may be more problematic for unbalanced designs. Third, the UKS test does not depend on whether the variance of Gaussian errors varies across individuals, while this kind of heteroscedasticity might affect type I and II error rates in ME analyses. Fourth, the UKS test do not need any assumption about the distribution of individual factor effects and is robust with respect to individual outliers, while violation of the normality assumption should bias the estimation of the random effect component and its CI in ME analyses.

## Discussion

### 1. Overview

Life and social sciences investigate systems whose behavior depends on multiple interacting components. Controlled experiments allow identification of these components by showing that individual factor effects either have a sample average significantly larger than expected from their inter-individual variability (*null average hypothesis*), or are larger than expected from the intra-individual residual variability (*global null hypothesis*). The second approach seems much more appropriate to life and social sciences than the first one. Indeed, it is more consistent with the scientific goals of most experiments – uncovering experimental factors that affect individual behavior rather than average behavior – and, in sharp contrast with the first approach, its power increases with inter-individual variability (Result Section part 1). However, the overwhelming majority of studies test for the “*null average hypothesis”* by using statistical tests such as t-tests, Anovas, linear regressions, logistic regression and other methods akin to general(ized) linear models. This is all the more damageable that the experimental effects that are the most likely to be overlooked are also likely to be the most informative. Indeed, when properly investigated, individually variable factors effects can shed more light into systemic processes than stereotypical effects. As recently stressed in various domains [27]–[33], we should embrace individual differences as a major source of knowledge rather than discard them as an uninteresting and disappointing nuisance.

From a methodological point of view, the new test of the *global null hypothesis* we propose has several important strengths that are worth emphasizing. First, at variance with alternative procedures (testing subject-factor interaction in RM Anovas, or meta-analytic methods for combining *p*-values), a highly significant effect in a single individual is never sufficient for the UKS test to reject the null hypothesis (Part 3). Second, at variance with RM Anovas, the procedure yields a highly reproducible outcome when factor effects are null or opposite in a part of the population (Part 2). Third, the procedure can also be applied with possible pretreatment when the assumptions of normality and homoscedasticity are not fully met in individual data (Part 4 to 6). Fourth, the UKS test can naturally be used in conjunction with non-parametric tests, if more suitable (Part 7). Fifth, it is more powerful than ME analyses when the number of individuals is smaller than 30 (Part 8). Last, the UKS procedure is easy to comprehend and therefore reduces the likelihood of errors in analysis or modeling.

### 2. Limits

A first limit regards the validity of the underlying statistical tests: these must meet their assumptions and must yield exact or well approximated statistics. The UKS test may yield inflated type I error rates when applied to approximate *p*-values of non-parametric statistics or maximum likelihood estimation (Part 7). Violation of the assumptions required by individual tests may lead to a similar inflation of false positives. We showed for simple designs that heteroscedasticity was not a threat, but that skewness in individual data is a serious difficulty. Nevertheless, type I error rate get back to nominal rate when individual data are log-transformed, at least in the simple Anova designs we investigated. Further investigations should extend the range of designs for which we understand the robustness of UKS. However, our investigations already makes the robustness of UKS procedure better understood than that of alternative methods for combining p-values [34] and, as far as we know, that of subject-factor interactions in RM Anovas and multilevel ME analyses. Finally, it should be noted that although individual Anovas with two or more factors require carrying out as many UKS tests as there are main effects and interactions of interest, corrections for multiple comparison are unnecessary. Indeed, it is traditionally considered that different F tests address conceptually distinct questions [23], and there is no more reason to apply corrections for multiple comparisons with the UKS test than with RM Anovas or multilevel mixed-effects analyses.

A second limit is the inference that “there is an effect in a non-negligible part of the population” when the UKS test is significant. While this may sound a weak conclusion, it must be clear that no stronger statement can be made on individual effects with non-significant sample average. Subject-effects interactions in RM Anovas and multilevel analyses assess the same *global null hypothesis* that there is no difference between individual and average effect in any individual. In contrast to UKS, interaction tests have null resistance and are probably as sensitive as meta-analytic methods to outlier individual *p*-values. In other words, we think that rejecting the *global null hypothesis* with the UKS test is more robust and reproducible than rejecting the same hypothesis with a meta-analytic method or a mixed-effects analysis. The price to pay for this higher robustness is that the former test is less powerful than the latter. As shown in Part 1 & 2, this trade-off remains in reasonable limits, since the power of the UKS test is comparable to that of RM Anovas.

A third limit concerns internal validity. As with any other test based on repeated-measures designs, experimenters are always at risk to confound the effects of experimental factors with those of learning, fatigue, individual maturation or lasting effects of treatments. This threat can be minimized by experimental designs orthogonalizing experimental factors and trial order. In addition, we consider that conclusions based on the UKS test should systematically be backed by the demonstration of no interactions between effects and trial order.

The last and probably most important limit concerns the required experimental design. The UKS test requires measurements to be repeated both across and within individuals (doubly repeated-measures designs): this is not always possible. As stressed by Friston and colleagues [5], learning experiments, as well as pharmacological studies when treatments have long-lasting effects, require random-effects analyses because their object is incompatible with repeated measurements.

### 3. UKS Test and Multilevel Mixed-effects Models

With respect to multilevel mixed-effects analyses, the UKS test has three advantages: it is of simple use; it is devoid of any assumption about the distribution of individual parameters; it can be used with small number of individuals. It may also provide more robust and reproducible results than tests of subject-factor interactions. However, multilevel analyses have a number of advantages with respect to UKS test. They can deal with missing data and correlation between successive trials. They are powerful methods for evidencing both inter-individual and intra-individual significant effects. They allow including second-level variables – individual characteristics – in the analysis. As emphasized by Baayen and colleagues [27], multilevel analyses have been developed to capture individual differences in a principled way and are the appropriate method for bringing individual differences into theories. However, they require extensive work and high statistical expertise for analyzing the data [25]. Moreover, the tests used by mixed-effect analyses have only asymptotic validity, which may cause difficulties in obtaining proper *p* values with small sample sizes [35], so that UKS is a safer method to evidence subject-factor interactions whenever population size is lower than 30 individuals (Part 8). In light of these observations, we believe that the simple and straightforward UKS test is a promising statistical tool that should help counterbalancing the bias of Anovas toward evidencing stereotypical effects. Its use in small exploratory designs can pave the way for later large-sample mixed-effects analyses. In this latter perspective, we think that papers based on the UKS test should include three key pieces of information in order to facilitate further investigations using multilevel models: (1) the percent of variance explained by each individual model, to allow assessing the size of the evidenced effects; (2) the distribution of individual parameters, to indicate how far they are from the Gaussian assumption required by multilevel models; (3) the correlations of all recorded individual characteristics with the fitted parameters, to help choosing the appropriate second-level variables in further investigations.

### 4. Conclusion

As regards the investigated objects, the scope of the UKS test is potentially very large. Virtually all experimental sciences study complex systems, and we cannot assess how often insight can be gained from comparing individual experimental effects to within-individual variability. With respect to simple repeated-measures designs analyzed with paired t-tests, RM Anovas or Ancovas, the UKS test offers a different perspective on data. The *null average hypothesis* is one way to evidence the effects of experimental factors. The *global null hypothesis* is another way, based on within- rather than between-individual variability. This different perspective may be determinant to highlight experimental effects that would be overlooked or misunderstood when across-individual average is compared to across-individual variability. In addition, papers based on the UKS test can set the stage for further investigations using multilevel analyses to model the relationship between experimental effects and individual characteristics. Finally, we are not without hoping that making the UKS test available may abate the inclination to force significant average effects out by discarding individuals, multiple testing [36] or other questionable practices [37].

## Methods

All Monte-Carlo simulations studies were carried out with MATLAB (The MathWorks, Natick, USA) except those involving multilevel mixed-effects analyses that were carried out with the R package nlme (The R Foundation for Statistical Computing, Vienna, Austria; http://www.r-project.org). MATLAB programs for parallelized computation of Anovas, repeated-measures Anovas and Kolmogorov-Smirnov tests were specially developed and controlled with respect to corresponding built-in functions of MATLAB or R. Specifically, the cumulative distribution function of the Kolmogorov-Smirnov statistics was estimated using the algorithm of the R function *ks.test*.

### Type II Errors in UKS Tests and RM Anovas

The comparison involved two stages. In the first one, we focused on how the median *p*-values yielded by both tests varied as a function of σ_int_ and σ_err_ for a two-way mixed design with 10 within-level trial repetitions, 2 levels in the fixed factor and 10 individuals. We computed the median probabilities yielded by RM Anovas and UKS tests for all combinations of 41 values for the standard deviation of the subject-factor interaction σ_int_ (from 0 to 4 by 0.1 steps) and 46 values for the trial-to-trial standard deviation σ_err_ (.001, .002, .005, .01, .02, .05, and from 0.1 to 4 by 0.1 steps). For each combination, we built 100000 random datasets of 200 trials by adding three values representing the factor’s effect, the individual variation of the factor’s effect and the trial-to-trial error. The first value was set to 1/√2 for level 1 and –1/√2 for level 2 so that *S*_eff_ was equal to 1 with *S*_eff_ defined by
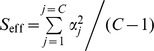
 and α_j_ is the value of the j^th^ of *C* effects. The second value was common to the 10 within-level repetitions of an individual and was drawn from a Gaussian distribution *N* (0, σ_int_). The third value was drawn from a Gaussian distribution with zero mean and a different standard deviation for each individual. These standard deviations were equal to 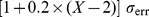, where *X* was randomly drawn from a gamma distribution with parameters *k* = 2 and θ = 1. This allowed intra-individual standard deviations to vary in a realistic way around σ_err_ (95% of values between 65% and 171% of σ_err_).

In the second stage, we investigated how the probabilities yielded by UKS tests and RM Anovas varied as a function of the number of individuals, factor levels and within-level repetitions. The effect of the number of individuals (*I* = 4, 5, 7, 10, 14 or 28) was systematically investigated in 27 studies that differed by the number of factor levels *C*, the number of repetitions *N*, or the ratio *VR* of the standard deviations of the subject-factor interaction and the trial-to-trial errors (*VR* = 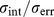 = 0.1, 0.2, 1/√10, 0.5, 1/√2 or 1). In two other studies, the number of factor levels was systematically varied (*C* = 2 to 6) for 2 combinations of *S*, *VR*, and *N*. In a final set of studies, the number of within-level repetitions (*N* = 2, 3, 5, 10, 20, 40 or 80) was systematically varied for 9 combinations of *S*, *C* and *VR*. In each of these 38 studies, we compute median probabilities from 10000 random datasets for 40 increasing (σ_err_, σ_int_) couples with a fixed *VR* ratio chosen so that UKS test and RM Anovas have commensurate type II error rates.

### Mixed Gaussian Distributions

We estimated the proportion *p* of significant outcome for UKS tests (.01 and .05 thresholds) and RM Anovas (.05) for a simple design where 10 individuals performed 8 trials in each of 2 different experimental conditions. Trial errors were drawn from a Gaussian distribution with parameters 0 and √8, so that the average of each experimental condition had unit variance. A constant value, randomly drawn *with replacement* from a set of 10 values, was added to all trials of the first experimental condition. In one simulation study, the set of 10 values contained one zero and nine values *d*. As a result, the individual differences between conditions’ averages were distributed according to a mixed Gaussian distribution including 10% of a Gaussian distribution with parameters (0, √2) and 90% of a Gaussian distribution with parameters (*d*, √2). In five other studies, the central parameters and proportions were as follows: [0, 20%; *d*, 80%]; [–*d*, 10%; *d*, 90%]; [0, 40%; *d*, 60%]; [–*d*, 20%; *d*, 80%]; [–*d*, 10%; 0; 20%; *d*, 70%]. In each simulation study, the value of *d* was varied from 0 to 8 by .2 steps. For each of these 41 *d* values, the proportion of significant tests was estimated from the outcome of 10000 Monte-Carlo runs.

### Type I Errors with Gaussian Distributions and Equal Variances

We draw random datasets from Gaussian distributions for each of 168 designs obtained by combinations of 2, 3, 4 or 5 levels in the factor, 4, 5, 7, 10, 20 or 40 individuals, and 2, 3, 5, 10, 20, 40 or 100 within-cell repetitions. We ran both UKS test and RM Anova on every random dataset to test whether the probability of wrongly highlighting an effect as significant was equal to the nominal 0.05 threshold. For computational considerations, the number of random datasets varied from 1000 for the largest datasets (20000 trials in the 5×40×100 combination) to 1250000 for the smallest (16 trials). Datasets were constructed as for type II error rate studies (see above) except that the factor effect and the inter-individual variability were set to zero. Note that setting σ_int_ to zero does not boil down to assume that the effects do not vary across individuals. The individual effects are expected to vary, but only because of trial-to-trial variability. If we had assumed that the SD of the interaction between the fixed and random factors were above zero, the UKS test would rightly have yielded more than 5% of significant results at the 0.05 threshold with a null average effect. Stating that the effect of an experimental factor genuinely varies across individuals is the same thing as affirming that the effect is not null in one individual, or even in all of them if its across-individual distribution is Gaussian.

### Type I Errors with Unequal Variances

We carried out a systematic investigation of the issue by drawing 216 samples of 60000 random datasets from Gaussian distributions. The large sample size (60000) was necessary to obtain reliable estimates of the type I error rates. The 216 samples were obtained by systematically combining the numbers of fixed-factor levels (2, 3 or 4), within-level repetitions (2, 3, 5, 10, 20, or 40), and individuals (5, 10 or 20) with 4 values of heteroscedasticity. The 4 values of heteroscedasticity were obtained by setting the ratio of the largest to the smallest variance to 2, 3, 4 or 8 (Figure 3, panel A) while maintaining the average variance unchanged. For the 4-level case, the ratio of variance of the two intermediate levels was set to the square root of the extremum ratio while their average was the same as that of the extreme levels (e.g.: 0.4, 2/3, 4/3 and 1.6). We varied the intra-individual variability σ_err_ across samples so that UKS test and RM Anova would have had a power around 60% if the effect size was 1 instead of 0 (measured values: 64.8±8.3% for the UKS test, 59.8±10.8% for RM Anova).

### Type I Errors with Non-Gaussian Distributions

We investigated the effects of violations of normality on type I errors for 84 designs obtained by combining the numbers of individuals (5, 7, 10 or 20), condition levels (2, 3 or 4), and within-level repetitions (2, 3, 5, 7, 10, 15, or 32). For each design, we drew 60000 random datasets from each of 16 different distributions: 3 standard Gaussian distributions used as baseline (3 control distributions allowed determining the limits between normal and abnormal rates on a large sample of 180000 datasets), 8 skewed distributions, and 5 distributions with outliers. The 8 skewed distributions included 2 gamma (θ = 1, k = 4; θ = 1, k = 2), 2 lognormal (μ = 0, σ = 1/√2; μ = 0, σ = 1), 2 Weibull (λ = 0.5, k = 1.2; λ = 1, k = 1.8), and 2 exponential distributions (β = 0.4; β = 1). The first of each pair is shown in Figure 3 (panel B). Weibull and exponential distributions were chosen for their use in fitting response time data [38]–[41], and the other distributions for investigating the effects of moderate skewness. Gaussian data were made positive by adding them a constant value equal to 1 minus the across-samples minimum of the data. This allowed performing Anovas on both the raw data and their natural logarithm. The 5 distributions with outliers were obtained by substituting abnormal values to fixed proportions of the data in datasets drawn from Gaussian distributions *N* (0,1). The first 2 distributions aimed at assessing the effect of outliers that could not be removed by standard procedures based on the 3 standard deviations threshold. In these distributions, 2.5% and 7.5% of the data was replaced by values drawn from Gaussian distribution *N*(2.5, 0.25). Therefore, 95% of outliers were expected to be between 2 and 3. The 3 other distributions included ‘removable’ outliers drawn from the distributions *N* (±3.5, 0.25). The first two included 2.5% and 7.5% of values centered on 3.5, and the last one 2.5% of values centered on –3.5 and 2.5% of values centered on +3.5. As for the other distributions, the data was made positive by adding a constant value equal to 1 minus the across-samples minimum of the data.

### Type I and Type II Errors with Individual Kruskal-Wallis Tests

The exact Kruskal-Wallis distributions were computed for designs with at most 21 trials by individual with a custom Matlab program. For type I errors, we used the same datasets as above for investigating the effects of violations of normality on type I errors, except those with more than 21 trials by individuals. For type II errors, we added fixed and random factor effects defined as in the first study (S_Eff_ = 1), except that σ_int_ was defined by 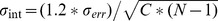 so as to enable RM Anovas and UKS test to have similar type II error levels (*C* and *N* are respectively the numbers of levels and within-level repetitions).

### Comparison with Multilevel Mixed-effect Models (Part 8)

We examined the estimates and CI of the factor effect and of the variance of errors, individual effects and individual averages for the full and restricted (null variance of the factor effect) ME models in 490 series of random datasets where the factor effect was set to zero. Confidence intervals were probed by means of two main indices: their size, and the percentage of CI including the theoretical value. CI size was defined as the ratio of the upper to lower CI limit and was compared to the ratio of the .975 to .025 quantile of random dataset sample variances in the series. Most series included 2000 random datasets (for computational reasons, the number of datasets was set to 1000 in designs with more than 3900 trials and 500 in designs with more than 7800 trials). Each series corresponded to a specific RM Anova design and a specific partial intraclass correlation coefficient (pICC). The 490 series were obtained by systematically combining 2 or 4 factor levels, 6, 8, 10, 15, 30, 50 or 100 individuals as population size, 3, 5, 10, 20 or 40 within-level repetitions, and 7 couples of standard deviations of the subject-factor interaction (σ_int_: 0.0, 0.2, 0.4, 0.6, 0.7, 1.2 or 4.0) and trial-to-trial errors (σ_err_: 0.9, 0.9, 0.9, 1.0, 1.0, 1.2 or 3.0 multiplied by the square root of the number of repetitions). The standard deviations together defined 7 ratios *R* = σ_int_^2^/(σ_err_^2^/*N*) from 0 to 16/9, which corresponded to approximate pICC = *R*/(1+*R*) from 0 to 0.64. In order to keep power similar across population sizes, the above values of σ_int_ and σ_err_, chosen for 10 individuals, were multiplied by the square root of *I*/10 where *I* is the number of individuals. Datasets were constructed as for the type I error rate studies of Part 4, except that we added to every data a value representing the individual variation of individuals’ grand mean. For every individual this value was drawn from a Gaussian distribution *N* (0, σ_subj_) where σ_subj_ was set to a high value (10 times the sum of σ_int_ and σ_err_) in order to avoid any problem of convergence caused by an estimation of inter-subject variance close to zero_._ Indeed, preliminary investigations showed that the results of ME analyses can be affected when σ_subj_^2^ is smaller than σ_int_^2^ divided by the number of factor levels (for the same reasons they are affected when σ_int_^2^ is smaller than σ_err_^2^ divided by the number of repetitions, see **Results S1**).

## Supporting Information

Table S1 The average percentage of failure in estimating the confidence intervals of variances using the R function *intervals* are displayed for 490 RM Anova designs. Each design is characterized by a number of individuals (6 to 100, column 1), a pICC value (0 to 0.64, column 2), a number of factor levels (2 or 4, top line) and a number of within-level repetitions (3 to 40, second line). The last column displays the grand mean across numbers of factor’s levels and repetitions. Averages are computed across 2000 datasets for most designs, and 500 or 1000 for the largest ones.
(DOC)

Table S2 Percentages of datasets with ill-defined confidence intervals for the factor effect variance σ_int_^2^, for the same 490 designs as in Table S1. Ill-defined confidence intervals are arbitrarily defined as CI with ratio of upper to lower limit over 100 times the ratio of the .975 to .025 quantile of random sample variances. Detailed investigations showed that percentages steadily increase as the ratio threshold decrease from 100 to 1 and decrease as the ratio threshold increase from 100 to the computer-dependent maximal value. Thus, the present pattern of results does not depend on the choice of a particular ratio threshold.
(DOC)

Table S3 The average percentage of datasets where a significantly non-null variance component σ_int_^2^ was evidenced are displayed for the same 490 RM Anova designs as in Table S1. For pICC equal to zero, the figures indicate the type I error rates, which are abnormally low (2% instead of 5%). For pICC above zero, the figures indicate the power.
(DOC)

Table S4 Type I error rates (%) in the restricted model for the same 490 designs as in Table S1. These percentages concern all datasets, rather than only those with type II errors in the full model. Note that the percentages vary as a function of pICC and number of condition levels and do not depend on the number of individuals.
(DOC)

Table S5 Percentages of datasets with no significant σ_int_^2^ in the full model (type II error when pICC>0) and type I error in the restricted model, for the same 490 designs as in Table S1. Note that for a low number of individuals (<15), the percentages increase with pICC, thus with ICC and the number of repetitions.
(DOC)

Table S6 Type I error rates (pICC = 0) and power (pICC>0) of UKS tests (%) for the same 490 designs as in Supplementary Table 1. Note that as for ME power (Table S3), the percentages increase with pICC and the number of individuals.
(DOC)

Table S7 Significance rates with UKS test minus significance rates for the random effect component of the full model in ME analyses, for the same 490 designs as in Table S1. The UKS test is more powerful than ME for evidencing non-null σ_int_^2^ component in designs with a small number of individuals or a low pICC.
(DOC)

Results S1 This section provides further results on the UKS test and additional detail on type II errors as a function of inter- and intra-individual variances (1), type I errors with violations of homoscedasticity in individual Anovas (2), type I errors with violations of normality in individual one-way Anovas (3), and Mixed-effects models analyses (4).
(DOC)
