# Supplementary material for: When One Size Does Not Fit All: A Simple Statistical Method to Deal with Across-Individual Variations of Effects
Source: PLoS One. 2012 Jun 18;7(6):e39059. doi: 10.1371/journal.pone.0039059 (PMC3377596; doi:10.1371/journal.pone.0039059)
Supplement: Table S1 — The average percentage of failure in estimating the confidence intervals of variances using the R function intervals are displayed for 490 RM Anova designs. Each design is characterized by a number of individuals (6 to 100, column 1), a pICC value (0 to 0.64, column 2), a number of factor levels (2 or 4, top line) and a number of within-level repetitions (3 to 40, second line). The last column displays the grand mean across numbers of factor’s levels and repetitions. Averages are computed across 2000 datasets for most designs, and 500 or 1000 for the largest ones. (DOC) [file pone.0039059.s001.doc]

| **Nb Cond (*C*)** | | **2** | | | | | | **4** | | | | | |  |
| --- | --- | --- | --- | --- | --- | --- | --- | --- | --- | --- | --- | --- | --- | --- |
| **Nb Repet (*N*)** | | **3** | **5** | **10** | **20** | **40** | **Mean** | **3** | **5** | **10** | **20** | **40** | **Mean** | **GdMn** |
| **Nb Indiv *I*** | **pICC** |  |  |  |  |  |  |  |  |  |  |  |  |  |
| **6** | **0.000** | 29 | 33 | 20 | 29 | 28 | **28** | 25 | 30 | 31 | 31 | 23 | **28** | 28 |
|  | **0.072** | 29 | 30 | 17 | 26 | 26 | **26** | 21 | 25 | 25 | 26 | 21 | **24** | 25 |
|  | **0.165** | 25 | 26 | 16 | 23 | 22 | **22** | 17 | 17 | 19 | 20 | 16 | **18** | 20 |
|  | **0.252** | 22 | 22 | 14 | 19 | 20 | **20** | 12 | 14 | 15 | 17 | 13 | **14** | 17 |
|  | **0.354** | 18 | 20 | 10 | 17 | 17 | **16** | 8 | 8 | 10 | 8 | 8 | **8** | 12 |
|  | **0.500** | 13 | 12 | 8 | 12 | 12 | **11** | 3 | 3 | 3 | 5 | 6 | **4** | 8 |
|  | **0.640** | 9 | 8 | 7 | 7 | 8 | **8** | 1 | 1 | 1 | 2 | 1 | **1** | 4 |
| **8** | **0.000** | 29 | 30 | 39 | 29 | 24 | **30** | 26 | 27 | 32 | 36 | 37 | **32** | 31 |
|  | **0.072** | 25 | 30 | 35 | 26 | 22 | **27** | 22 | 21 | 28 | 31 | 33 | **27** | 27 |
|  | **0.165** | 23 | 27 | 28 | 23 | 19 | **24** | 17 | 15 | 21 | 24 | 23 | **20** | 22 |
|  | **0.252** | 20 | 21 | 22 | 19 | 16 | **20** | 12 | 11 | 14 | 14 | 17 | **13** | 17 |
|  | **0.354** | 15 | 16 | 19 | 14 | 12 | **15** | 8 | 6 | 8 | 9 | 11 | **8** | 12 |
|  | **0.500** | 8 | 10 | 11 | 8 | 10 | **9** | 2 | 2 | 2 | 4 | 9 | **4** | 7 |
|  | **0.640** | 4 | 6 | 6 | 4 | 5 | **5** | 0 | 0 | 0 | 0 | 1 | **0** | 3 |
| **10** | **0.000** | 27 | 26 | 38 | 36 | 37 | **33** | 28 | 27 | 32 | 29 | 33 | **30** | 31 |
|  | **0.072** | 26 | 25 | 33 | 34 | 33 | **30** | 22 | 21 | 26 | 22 | 27 | **23** | 27 |
|  | **0.165** | 21 | 21 | 28 | 29 | 30 | **26** | 15 | 16 | 19 | 16 | 18 | **17** | 21 |
|  | **0.252** | 17 | 18 | 24 | 23 | 26 | **22** | 10 | 10 | 12 | 12 | 13 | **11** | 16 |
|  | **0.354** | 13 | 11 | 18 | 18 | 21 | **16** | 5 | 4 | 4 | 5 | 11 | **6** | 11 |
|  | **0.500** | 8 | 7 | 10 | 11 | 13 | **10** | 1 | 1 | 2 | 4 | 8 | **3** | 6 |
|  | **0.640** | 3 | 3 | 4 | 6 | 5 | **4** | 0 | 0 | 0 | 0 | 0 | **0** | 2 |
| **15** | **0.000** | 25 | 31 | 31 | 28 | 26 | **28** | 29 | 29 | 26 | 29 | 33 | **29** | 29 |
|  | **0.072** | 22 | 27 | 26 | 27 | 23 | **25** | 22 | 22 | 20 | 20 | 25 | **22** | 23 |
|  | **0.165** | 18 | 21 | 21 | 20 | 20 | **20** | 14 | 14 | 12 | 14 | 16 | **14** | 17 |
|  | **0.252** | 16 | 16 | 16 | 17 | 20 | **17** | 8 | 9 | 7 | 8 | 13 | **9** | 13 |
|  | **0.354** | 9 | 11 | 10 | 13 | 16 | **12** | 3 | 2 | 2 | 5 | 11 | **5** | 8 |
|  | **0.500** | 3 | 5 | 5 | 7 | 9 | **6** | 0 | 0 | 1 | 2 | 5 | **2** | 4 |
|  | **0.640** | 1 | 2 | 2 | 1 | 2 | **1** | 0 | 0 | 0 | 0 | 0 | **0** | 1 |
| **30** | **0.000** | 29 | 28 | 30 | 28 | 34 | **30** | 27 | 25 | 28 | 30 | 25 | **27** | 29 |
|  | **0.072** | 24 | 23 | 24 | 25 | 28 | **25** | 19 | 18 | 20 | 21 | 17 | **19** | 22 |
|  | **0.165** | 15 | 17 | 20 | 18 | 23 | **19** | 10 | 9 | 10 | 14 | 12 | **11** | 15 |
|  | **0.252** | 11 | 11 | 13 | 15 | 18 | **14** | 4 | 5 | 6 | 13 | 9 | **7** | 10 |
|  | **0.354** | 4 | 6 | 7 | 9 | 11 | **8** | 2 | 1 | 3 | 7 | 5 | **4** | 6 |
|  | **0.500** | 2 | 2 | 2 | 2 | 3 | **2** | 0 | 0 | 0 | 0 | 0 | **0** | 1 |
|  | **0.640** | 0 | 0 | 0 | 0 | 0 | **0** | 0 | 0 | 0 | 0 | 0 | **0** | 0 |
| **50** | **0.000** | 29 | 33 | 28 | 46 | 37 | **35** | 28 | 29 | 29 | 29 | 58 | **34** | 35 |
|  | **0.072** | 23 | 28 | 23 | 38 | 27 | **28** | 18 | 22 | 21 | 21 | 55 | **28** | 28 |
|  | **0.165** | 16 | 20 | 17 | 29 | 24 | **21** | 10 | 12 | 13 | 13 | 46 | **19** | 20 |
|  | **0.252** | 10 | 12 | 9 | 20 | 15 | **13** | 5 | 5 | 8 | 9 | 28 | **11** | 12 |
|  | **0.354** | 4 | 5 | 4 | 9 | 8 | **6** | 1 | 1 | 2 | 1 | 4 | **2** | 4 |
|  | **0.500** | 0 | 0 | 0 | 1 | 1 | **1** | 0 | 0 | 0 | 0 | 1 | **0** | 0 |
|  | **0.640** | 0 | 0 | 0 | 0 | 0 | **0** | 0 | 0 | 1 | 1 | 0 | **0** | 0 |
| **100** | **0.000** | 36 | 36 | 44 | 38 | 60 | **43** | 43 | 44 | 40 | 58 | 62 | **49** | 46 |
|  | **0.072** | 29 | 30 | 36 | 30 | 50 | **35** | 34 | 35 | 34 | 52 | 59 | **43** | 39 |
|  | **0.165** | 16 | 18 | 23 | 18 | 32 | **21** | 16 | 15 | 15 | 25 | 34 | **21** | 21 |
|  | **0.252** | 6 | 7 | 8 | 7 | 14 | **8** | 2 | 2 | 2 | 4 | 4 | **3** | 6 |
|  | **0.354** | 1 | 1 | 1 | 1 | 2 | **1** | 0 | 0 | 0 | 0 | 0 | **0** | 1 |
|  | **0.500** | 0 | 0 | 0 | 0 | 0 | **0** | 0 | 0 | 0 | 0 | 0 | **0** | 0 |
|  | **0.640** | 0 | 0 | 0 | 0 | 1 | **0** | 0 | 0 | 0 | 0 | 0 | **0** | 0 |

**Table S1: Percentage of failures in estimating the factor effect variance CI.**
