# Supplementary material for: When One Size Does Not Fit All: A Simple Statistical Method to Deal with Across-Individual Variations of Effects
Source: PLoS One. 2012 Jun 18;7(6):e39059. doi: 10.1371/journal.pone.0039059 (PMC3377596; doi:10.1371/journal.pone.0039059)
Supplement: Table S2 — Percentages of datasets with ill-defined confidence intervals for the factor effect variance σint 2, for the same 490 designs as in Table S1. Ill-defined confidence intervals are arbitrarily defined as CI with ratio of upper to lower limit over 100 times the ratio of the .975 to .025 quantile of random sample variances. Detailed investigations showed that percentages steadily increase as the ratio threshold decrease from 100 to 1 and decrease as the ratio threshold increase from 100 to the computer-dependent maximal value. Thus, the present pattern of results does not depend on the choice of a particular ratio threshold. (DOC) [file pone.0039059.s002.doc]

| **Nb Cond (*C*)** | | **2** | | | | | | **4** | | | | | |  |
| --- | --- | --- | --- | --- | --- | --- | --- | --- | --- | --- | --- | --- | --- | --- |
| **Nb Repet (*N*)** | | **3** | **5** | **10** | **20** | **40** | **Mean** | **3** | **5** | **10** | **20** | **40** | **Mean** | **GdMn** |
| **Nb Indiv. *I*** | **pICC** |  |  |  |  |  |  |  |  |  |  |  |  |  |
|  | **0.000** | 27 | 30 | 30 | 27 | 28 | **29** | 26 | 24 | 23 | 26 | 25 | **25** | 27 |
| **6** | **0.072** | 25 | 26 | 26 | 26 | 24 | **25** | 22 | 22 | 22 | 22 | 24 | **22** | 24 |
|  | **0.165** | 23 | 22 | 22 | 22 | 20 | **22** | 17 | 17 | 18 | 17 | 18 | **17** | 20 |
|  | **0.252** | 19 | 19 | 20 | 19 | 19 | **19** | 13 | 14 | 14 | 13 | 12 | **13** | 16 |
|  | **0.354** | 16 | 15 | 16 | 16 | 15 | **15** | 10 | 10 | 9 | 9 | 9 | **9** | 12 |
|  | **0.500** | 11 | 11 | 11 | 12 | 11 | **11** | 5 | 4 | 4 | 3 | 4 | **4** | 8 |
|  | **0.640** | 6 | 6 | 5 | 5 | 3 | **5** | 2 | 1 | 1 | 1 | 1 | **1** | 3 |
| **8** | **0.000** | 27 | 28 | 27 | 28 | 24 | **27** | 26 | 26 | 26 | 23 | 25 | **25** | 26 |
|  | **0.072** | 25 | 22 | 22 | 27 | 23 | **24** | 22 | 20 | 23 | 19 | 21 | **21** | 23 |
|  | **0.165** | 21 | 21 | 22 | 22 | 20 | **21** | 17 | 16 | 18 | 17 | 16 | **17** | 19 |
|  | **0.252** | 17 | 19 | 16 | 18 | 17 | **17** | 11 | 13 | 12 | 13 | 12 | **12** | 15 |
|  | **0.354** | 14 | 13 | 13 | 13 | 14 | **13** | 8 | 8 | 8 | 6 | 7 | **7** | 10 |
|  | **0.500** | 10 | 9 | 10 | 9 | 9 | **9** | 3 | 3 | 3 | 2 | 3 | **3** | 6 |
|  | **0.640** | 4 | 3 | 3 | 2 | 1 | **3** | 1 | 1 | 0 | 0 | 0 | **0** | 1 |
| **10** | **0.000** | 25 | 28 | 26 | 26 | 28 | **26** | 26 | 26 | 24 | 26 | 25 | **25** | 26 |
|  | **0.072** | 24 | 25 | 24 | 27 | 25 | **25** | 19 | 19 | 22 | 22 | 19 | **20** | 23 |
|  | **0.165** | 23 | 19 | 20 | 19 | 23 | **21** | 15 | 15 | 17 | 15 | 14 | **15** | 18 |
|  | **0.252** | 18 | 17 | 17 | 18 | 18 | **18** | 11 | 10 | 9 | 11 | 10 | **10** | 14 |
|  | **0.354** | 12 | 13 | 13 | 13 | 13 | **13** | 6 | 7 | 5 | 6 | 6 | **6** | 9 |
|  | **0.500** | 8 | 8 | 7 | 7 | 7 | **7** | 2 | 2 | 1 | 2 | 2 | **2** | 5 |
|  | **0.640** | 2 | 2 | 2 | 1 | 1 | **2** | 0 | 0 | 0 | 0 | 0 | **0** | 1 |
| **15** | **0.000** | 25 | 25 | 26 | 27 | 25 | **26** | 24 | 24 | 25 | 25 | 26 | **25** | 25 |
|  | **0.072** | 23 | 21 | 21 | 22 | 21 | **22** | 20 | 20 | 19 | 20 | 19 | **20** | 21 |
|  | **0.165** | 17 | 19 | 18 | 19 | 16 | **18** | 14 | 12 | 12 | 13 | 11 | **13** | 15 |
|  | **0.252** | 15 | 15 | 13 | 13 | 15 | **14** | 9 | 8 | 8 | 9 | 7 | **8** | 11 |
|  | **0.354** | 10 | 9 | 10 | 8 | 10 | **10** | 5 | 4 | 3 | 3 | 4 | **4** | 7 |
|  | **0.500** | 5 | 5 | 5 | 4 | 4 | **5** | 1 | 0 | 0 | 0 | 0 | **0** | 3 |
|  | **0.640** | 1 | 1 | 1 | 1 | 0 | **1** | 0 | 0 | 0 | 0 | 0 | **0** | 0 |
| **30** | **0.000** | 24 | 23 | 27 | 25 | 27 | **25** | 23 | 22 | 22 | 24 | 21 | **23** | 24 |
|  | **0.072** | 19 | 21 | 18 | 18 | 21 | **20** | 18 | 19 | 16 | 15 | 14 | **16** | 18 |
|  | **0.165** | 14 | 15 | 14 | 15 | 16 | **15** | 10 | 9 | 10 | 9 | 9 | **9** | 12 |
|  | **0.252** | 11 | 9 | 10 | 9 | 9 | **10** | 5 | 4 | 4 | 3 | 2 | **4** | 7 |
|  | **0.354** | 7 | 6 | 6 | 6 | 6 | **6** | 1 | 1 | 1 | 1 | 0 | **1** | 3 |
|  | **0.500** | 2 | 2 | 2 | 1 | 1 | **1** | 0 | 0 | 0 | 0 | 0 | **0** | 1 |
|  | **0.640** | 0 | 0 | 0 | 0 | 0 | **0** | 0 | 0 | 0 | 0 | 0 | **0** | 0 |
| **50** | **0.000** | 25 | 26 | 21 | 32 | 25 | **26** | 22 | 21 | 23 | 21 | 28 | **23** | 24 |
|  | **0.072** | 22 | 24 | 19 | 22 | 15 | **21** | 15 | 16 | 15 | 13 | 24 | **17** | 19 |
|  | **0.165** | 13 | 15 | 12 | 14 | 10 | **13** | 7 | 7 | 6 | 6 | 10 | **7** | 10 |
|  | **0.252** | 8 | 8 | 6 | 8 | 4 | **7** | 2 | 2 | 1 | 1 | 1 | **1** | 4 |
|  | **0.354** | 4 | 3 | 3 | 3 | 1 | **3** | 1 | 0 | 0 | 0 | 0 | **0** | 1 |
|  | **0.500** | 0 | 0 | 0 | 0 | 0 | **0** | 0 | 0 | 0 | 0 | 0 | **0** | 0 |
|  | **0.640** | 0 | 0 | 0 | 0 | 0 | **0** | 0 | 0 | 0 | 0 | 0 | **0** | 0 |
| **100** | **0.000** | 25 | 21 | 20 | 13 | 9 | **18** | 15 | 20 | 17 | 16 | 9 | **15** | 16 |
|  | **0.072** | 14 | 14 | 14 | 10 | 8 | **12** | 9 | 8 | 8 | 12 | 5 | **8** | 10 |
|  | **0.165** | 7 | 7 | 7 | 6 | 3 | **6** | 3 | 2 | 1 | 3 | 1 | **2** | 4 |
|  | **0.252** | 2 | 1 | 1 | 1 | 0 | **1** | 0 | 0 | 0 | 0 | 0 | **0** | 1 |
|  | **0.354** | 0 | 0 | 0 | 0 | 0 | **0** | 0 | 0 | 0 | 0 | 0 | **0** | 0 |
|  | **0.500** | 0 | 0 | 0 | 0 | 0 | **0** | 0 | 0 | 0 | 0 | 0 | **0** | 0 |
|  | **0.640** | 0 | 0 | 0 | 0 | 0 | **0** | 0 | 0 | 0 | 0 | 0 | **0** | 0 |

**Table S2: Percentage of datasets with ill-defined confidence intervals for the factor effect variance σ_int_^2^.**
