# Supplementary material for: When One Size Does Not Fit All: A Simple Statistical Method to Deal with Across-Individual Variations of Effects
Source: PLoS One. 2012 Jun 18;7(6):e39059. doi: 10.1371/journal.pone.0039059 (PMC3377596; doi:10.1371/journal.pone.0039059)
Supplement: Table S3 — The average percentage of datasets where a significantly non-null variance component σint 2 was evidenced are displayed for the same 490 RM Anova designs as in Table S1. For pICC equal to zero, the figures indicate the type I error rates, which are abnormally low (2% instead of 5%). For pICC above zero, the figures indicate the power. (DOC) [file pone.0039059.s003.doc]

| **Nb Cond (*C*)** | | **2** | | | | | | **4** | | | | | |  |
| --- | --- | --- | --- | --- | --- | --- | --- | --- | --- | --- | --- | --- | --- | --- |
| **Nb Repet (*N*)** | | **3** | **5** | **10** | **20** | **40** | **Mean** | **3** | **5** | **10** | **20** | **40** | **Mean** | **GdMn** |
| **Nb Indiv *I*** | **pICC** |  |  |  |  |  |  |  |  |  |  |  |  |  |
| **6** | **0.000** | 2 | 2 | 2 | 2 | 2 | **2** | 1 | 2 | 2 | 2 | 2 | **2** | 2 |
|  | **0.072** | 2 | 3 | 2 | 3 | 2 | **2** | 3 | 3 | 3 | 3 | 3 | **3** | 3 |
|  | **0.165** | 4 | 4 | 4 | 4 | 4 | **4** | 7 | 6 | 7 | 7 | 8 | **7** | 5 |
|  | **0.252** | 6 | 6 | 5 | 6 | 5 | **6** | 10 | 10 | 12 | 12 | 10 | **11** | 8 |
|  | **0.354** | 9 | 10 | 10 | 11 | 10 | **10** | 18 | 22 | 22 | 24 | 25 | **22** | 16 |
|  | **0.500** | 18 | 20 | 21 | 21 | 23 | **21** | 40 | 45 | 49 | 49 | 52 | **47** | 34 |
|  | **0.640** | 34 | 35 | 39 | 41 | 43 | **38** | 70 | 73 | 79 | 79 | 80 | **76** | 57 |
| **8** | **0.000** | 2 | 2 | 1 | 2 | 2 | **2** | 2 | 2 | 2 | 2 | 2 | **2** | 2 |
|  | **0.072** | 2 | 2 | 2 | 2 | 3 | **2** | 2 | 5 | 4 | 4 | 4 | **4** | 3 |
|  | **0.165** | 5 | 4 | 4 | 5 | 5 | **5** | 6 | 8 | 7 | 8 | 8 | **8** | 6 |
|  | **0.252** | 8 | 8 | 8 | 7 | 6 | **7** | 13 | 14 | 16 | 15 | 14 | **15** | 11 |
|  | **0.354** | 12 | 12 | 12 | 13 | 14 | **13** | 25 | 27 | 30 | 31 | 30 | **29** | 21 |
|  | **0.500** | 24 | 25 | 27 | 28 | 28 | **27** | 52 | 58 | 59 | 64 | 63 | **59** | 43 |
|  | **0.640** | 44 | 48 | 50 | 50 | 53 | **49** | 82 | 87 | 90 | 90 | 90 | **88** | 68 |
| **10** | **0.000** | 2 | 2 | 2 | 2 | 1 | **2** | 2 | 2 | 2 | 2 | 2 | **2** | 2 |
|  | **0.072** | 3 | 3 | 3 | 3 | 4 | **3** | 5 | 4 | 4 | 4 | 5 | **4** | 4 |
|  | **0.165** | 5 | 5 | 5 | 6 | 5 | **5** | 9 | 9 | 10 | 9 | 9 | **9** | 7 |
|  | **0.252** | 8 | 7 | 9 | 8 | 9 | **8** | 13 | 17 | 18 | 19 | 20 | **18** | 13 |
|  | **0.354** | 14 | 13 | 16 | 15 | 16 | **15** | 30 | 32 | 36 | 38 | 37 | **35** | 25 |
|  | **0.500** | 28 | 32 | 32 | 33 | 34 | **32** | 58 | 66 | 69 | 72 | 73 | **68** | 50 |
|  | **0.640** | 54 | 57 | 59 | 58 | 59 | **58** | 89 | 92 | 95 | 95 | 96 | **93** | 75 |
| **15** | **0.000** | 3 | 2 | 3 | 2 | 2 | **2** | 3 | 2 | 3 | 2 | 2 | **2** | 2 |
|  | **0.072** | 3 | 3 | 4 | 3 | 3 | **3** | 5 | 4 | 4 | 5 | 5 | **5** | 4 |
|  | **0.165** | 6 | 5 | 6 | 6 | 7 | **6** | 11 | 12 | 13 | 11 | 14 | **12** | 9 |
|  | **0.252** | 11 | 11 | 11 | 12 | 11 | **11** | 21 | 24 | 24 | 26 | 27 | **25** | 18 |
|  | **0.354** | 20 | 20 | 21 | 23 | 22 | **21** | 42 | 47 | 52 | 51 | 52 | **49** | 35 |
|  | **0.500** | 38 | 43 | 44 | 50 | 49 | **45** | 79 | 85 | 87 | 88 | 87 | **85** | 65 |
|  | **0.640** | 69 | 74 | 75 | 77 | 77 | **74** | 97 | 99 | 99 | 99 | 99 | **99** | 87 |
| **30** | **0.000** | 2 | 2 | 2 | 2 | 2 | **2** | 2 | 3 | 2 | 3 | 2 | **2** | 2 |
|  | **0.072** | 4 | 4 | 4 | 4 | 4 | **4** | 6 | 7 | 7 | 6 | 7 | **6** | 5 |
|  | **0.165** | 10 | 9 | 10 | 10 | 10 | **10** | 18 | 19 | 21 | 22 | 22 | **20** | 15 |
|  | **0.252** | 17 | 20 | 20 | 22 | 22 | **20** | 38 | 42 | 46 | 47 | 47 | **44** | 32 |
|  | **0.354** | 33 | 37 | 41 | 39 | 40 | **38** | 69 | 77 | 81 | 82 | 82 | **78** | 58 |
|  | **0.500** | 69 | 70 | 73 | 74 | 75 | **72** | 98 | 98 | 99 | 99 | 100 | **99** | 85 |
|  | **0.640** | 93 | 95 | 96 | 96 | 97 | **95** | 100 | 100 | 100 | 100 | 100 | **100** | 98 |
| **50** | **0.000** | 2 | 2 | 2 | 2 | 3 | **2** | 2 | 2 | 3 | 3 | 2 | **2** | 2 |
|  | **0.072** | 5 | 4 | 5 | 6 | 6 | **5** | 8 | 8 | 9 | 7 | 8 | **8** | 7 |
|  | **0.165** | 12 | 13 | 14 | 13 | 14 | **13** | 27 | 30 | 33 | 32 | 38 | **32** | 23 |
|  | **0.252** | 27 | 27 | 31 | 31 | 30 | **29** | 58 | 64 | 66 | 67 | 74 | **66** | 47 |
|  | **0.354** | 51 | 55 | 59 | 58 | 59 | **56** | 90 | 92 | 94 | 96 | 96 | **94** | 75 |
|  | **0.500** | 86 | 89 | 91 | 92 | 92 | **90** | 100 | 100 | 100 | 100 | 99 | **100** | 95 |
|  | **0.640** | 99 | 100 | 100 | 100 | 100 | **100** | 100 | 100 | 99 | 99 | 100 | **100** | 100 |
| **100** | **0.000** | 2 | 2 | 2 | 2 | 4 | **3** | 3 | 3 | 2 | 2 | 2 | **2** | 2 |
|  | **0.072** | 7 | 6 | 7 | 7 | 8 | **7** | 11 | 12 | 12 | 15 | 15 | **13** | 10 |
|  | **0.165** | 22 | 21 | 24 | 25 | 26 | **24** | 47 | 52 | 57 | 59 | 59 | **55** | 39 |
|  | **0.252** | 46 | 50 | 54 | 50 | 54 | **51** | 86 | 89 | 92 | 91 | 93 | **90** | 71 |
|  | **0.354** | 81 | 83 | 86 | 86 | 91 | **85** | 100 | 100 | 100 | 100 | 100 | **100** | 93 |
|  | **0.500** | 99 | 100 | 100 | 100 | 99 | **99** | 100 | 100 | 100 | 100 | 100 | **100** | 100 |
|  | **0.640** | 100 | 100 | 100 | 100 | 99 | **100** | 100 | 100 | 100 | 100 | 100 | **100** | 100 |

**Table S3: Power rates (%) for evidencing non-zero factor effect variance σ_int_^2^**.
