# Supplementary material for: When One Size Does Not Fit All: A Simple Statistical Method to Deal with Across-Individual Variations of Effects
Source: PLoS One. 2012 Jun 18;7(6):e39059. doi: 10.1371/journal.pone.0039059 (PMC3377596; doi:10.1371/journal.pone.0039059)
Supplement: Table S4 — Type I error rates (%) in the restricted model for the same 490 designs as in Table S1. These percentages concern all datasets, rather than only those with type II errors in the full model. Note that the percentages vary as a function of pICC and number of condition levels and do not depend on the number of individuals. (DOC) [file pone.0039059.s004.doc]

| **Nb Cond (*C*)** | | **2** | | | | | | **12** | | | | | |  |
| --- | --- | --- | --- | --- | --- | --- | --- | --- | --- | --- | --- | --- | --- | --- |
| **Nb Repet (*N*)** | | **3** | **5** | **10** | **20** | **40** | **Mean** | **3** | **5** | **10** | **20** | **40** | **Mean** | **GdMn** |
| **Nb Indiv *I*** | **pICC** |  |  |  |  |  |  |  |  |  |  |  |  |  |
| **6** | **0.000** | 5 | 5 | 4 | 4 | 5 | **5** | 5 | 5 | 6 | 5 | 5 | **5** | 5 |
|  | **0.072** | 6 | 6 | 6 | 6 | 6 | **6** | 6 | 6 | 6 | 7 | 5 | **6** | 6 |
|  | **0.165** | 6 | 7 | 7 | 8 | 8 | **7** | 9 | 8 | 9 | 9 | 9 | **9** | 8 |
|  | **0.252** | 7 | 9 | 9 | 8 | 8 | **8** | 11 | 10 | 10 | 12 | 12 | **11** | 10 |
|  | **0.354** | 10 | 10 | 10 | 11 | 11 | **10** | 13 | 13 | 16 | 16 | 16 | **15** | 13 |
|  | **0.500** | 13 | 15 | 15 | 16 | 17 | **15** | 18 | 21 | 26 | 25 | 26 | **23** | 19 |
|  | **0.640** | 19 | 20 | 21 | 23 | 23 | **21** | 28 | 32 | 37 | 38 | 43 | **36** | 28 |
| **8** | **0.000** | 6 | 6 | 5 | 6 | 5 | **5** | 5 | 5 | 5 | 5 | 5 | **5** | 5 |
|  | **0.072** | 5 | 6 | 6 | 7 | 6 | **6** | 5 | 7 | 6 | 6 | 6 | **6** | 6 |
|  | **0.165** | 7 | 8 | 6 | 8 | 7 | **7** | 8 | 10 | 9 | 8 | 8 | **9** | 8 |
|  | **0.252** | 9 | 8 | 9 | 10 | 10 | **9** | 9 | 9 | 11 | 10 | 12 | **10** | 10 |
|  | **0.354** | 9 | 11 | 11 | 12 | 10 | **11** | 11 | 14 | 15 | 14 | 17 | **14** | 12 |
|  | **0.500** | 12 | 14 | 16 | 15 | 16 | **15** | 19 | 22 | 25 | 25 | 26 | **23** | 19 |
|  | **0.640** | 16 | 21 | 21 | 22 | 24 | **21** | 26 | 30 | 37 | 38 | 40 | **34** | 27 |
| **10** | **0.000** | 6 | 5 | 5 | 6 | 6 | **5** | 5 | 4 | 5 | 5 | 4 | **5** | 5 |
|  | **0.072** | 6 | 6 | 6 | 6 | 6 | **6** | 5 | 6 | 6 | 6 | 6 | **6** | 6 |
|  | **0.165** | 8 | 8 | 7 | 7 | 8 | **8** | 8 | 8 | 9 | 9 | 9 | **9** | 8 |
|  | **0.252** | 8 | 8 | 8 | 9 | 8 | **8** | 9 | 11 | 11 | 12 | 11 | **11** | 10 |
|  | **0.354** | 10 | 10 | 10 | 11 | 11 | **10** | 13 | 15 | 15 | 16 | 16 | **15** | 13 |
|  | **0.500** | 13 | 14 | 15 | 16 | 17 | **15** | 18 | 21 | 24 | 26 | 25 | **23** | 19 |
|  | **0.640** | 18 | 21 | 21 | 22 | 24 | **21** | 25 | 31 | 36 | 40 | 38 | **34** | 27 |
| **15** | **0.000** | 5 | 4 | 5 | 5 | 5 | **5** | 6 | 4 | 5 | 5 | 5 | **5** | 5 |
|  | **0.072** | 6 | 5 | 6 | 6 | 7 | **6** | 7 | 6 | 6 | 7 | 6 | **6** | 6 |
|  | **0.165** | 8 | 8 | 6 | 7 | 6 | **7** | 8 | 8 | 7 | 10 | 9 | **8** | 8 |
|  | **0.252** | 8 | 8 | 8 | 9 | 8 | **8** | 9 | 11 | 11 | 11 | 11 | **11** | 9 |
|  | **0.354** | 10 | 10 | 12 | 10 | 12 | **11** | 13 | 13 | 15 | 17 | 18 | **15** | 13 |
|  | **0.500** | 13 | 15 | 15 | 15 | 16 | **15** | 18 | 22 | 25 | 23 | 27 | **23** | 19 |
|  | **0.640** | 18 | 19 | 23 | 21 | 23 | **21** | 25 | 31 | 37 | 40 | 40 | **35** | 28 |
| **30** | **0.000** | 4 | 5 | 5 | 5 | 6 | **5** | 6 | 6 | 5 | 5 | 5 | **5** | 5 |
|  | **0.072** | 6 | 6 | 6 | 5 | 6 | **6** | 6 | 7 | 6 | 6 | 5 | **6** | 6 |
|  | **0.165** | 7 | 7 | 7 | 7 | 8 | **7** | 7 | 8 | 9 | 8 | 8 | **8** | 8 |
|  | **0.252** | 8 | 7 | 8 | 7 | 9 | **8** | 10 | 10 | 10 | 10 | 11 | **10** | 9 |
|  | **0.354** | 9 | 12 | 10 | 12 | 12 | **11** | 12 | 14 | 14 | 15 | 15 | **14** | 13 |
|  | **0.500** | 14 | 15 | 15 | 17 | 16 | **16** | 16 | 22 | 24 | 27 | 28 | **23** | 20 |
|  | **0.640** | 18 | 19 | 24 | 22 | 25 | **22** | 24 | 30 | 38 | 39 | 39 | **34** | 28 |
| **50** | **0.000** | 5 | 4 | 6 | 5 | 5 | **5** | 5 | 6 | 4 | 4 | 5 | **5** | 5 |
|  | **0.072** | 5 | 6 | 6 | 6 | 6 | **6** | 6 | 5 | 6 | 6 | 6 | **6** | 6 |
|  | **0.165** | 7 | 8 | 7 | 7 | 6 | **7** | 7 | 9 | 8 | 7 | 8 | **8** | 8 |
|  | **0.252** | 8 | 9 | 9 | 9 | 9 | **9** | 9 | 11 | 11 | 12 | 11 | **11** | 10 |
|  | **0.354** | 11 | 10 | 11 | 12 | 12 | **11** | 14 | 13 | 15 | 16 | 18 | **15** | 13 |
|  | **0.500** | 13 | 14 | 16 | 17 | 15 | **15** | 17 | 23 | 25 | 26 | 26 | **23** | 19 |
|  | **0.640** | 17 | 20 | 21 | 24 | 25 | **21** | 22 | 32 | 35 | 38 | 40 | **33** | 27 |
| **100** | **0.000** | 5 | 5 | 5 | 4 | 6 | **5** | 5 | 5 | 4 | 7 | 5 | **5** | 5 |
|  | **0.072** | 6 | 5 | 6 | 6 | 5 | **6** | 5 | 6 | 7 | 5 | 6 | **6** | 6 |
|  | **0.165** | 7 | 8 | 7 | 8 | 8 | **8** | 7 | 8 | 8 | 10 | 8 | **8** | 8 |
|  | **0.252** | 7 | 9 | 8 | 9 | 8 | **8** | 10 | 11 | 12 | 14 | 11 | **12** | 10 |
|  | **0.354** | 10 | 11 | 11 | 11 | 11 | **11** | 13 | 13 | 16 | 15 | 16 | **14** | 13 |
|  | **0.500** | 13 | 14 | 16 | 15 | 18 | **15** | 17 | 22 | 26 | 25 | 27 | **23** | 19 |
|  | **0.640** | 19 | 20 | 22 | 21 | 21 | **20** | 23 | 31 | 39 | 43 | 38 | **35** | 28 |

**Table S4: Type I error rates (%) in the restricted model.**
