# Supplementary material for: When One Size Does Not Fit All: A Simple Statistical Method to Deal with Across-Individual Variations of Effects
Source: PLoS One. 2012 Jun 18;7(6):e39059. doi: 10.1371/journal.pone.0039059 (PMC3377596; doi:10.1371/journal.pone.0039059)
Supplement: Table S5 — Percentages of datasets with no significant σint 2 in the full model (type II error when pICC>0) and type I error in the restricted model, for the same 490 designs as in Table S1. Note that for a low number of individuals (<15), the percentages increase with pICC, thus with ICC and the number of repetitions. (DOC) [file pone.0039059.s005.doc]

| **Nb Cond (*C*)** | | **2** | | | | | | **4** | | | | | |  |
| --- | --- | --- | --- | --- | --- | --- | --- | --- | --- | --- | --- | --- | --- | --- |
| **Nb Repet (*N*)** | | **3** | **5** | **10** | **20** | **40** | **Mean** | **3** | **5** | **10** | **20** | **40** | **Mean** | **GdMn** |
| **Nb Indiv *I*** | **pICC** |  |  |  |  |  |  |  |  |  |  |  |  |  |
| **6** | **0.000** | 4.4 | 4.9 | 4.4 | 4.3 | 5.2 | **4.6** | 5.1 | 4.5 | 6.1 | 4.7 | 5.1 | **5.1** | 4.9 |
|  | **0.072** | 5.7 | 5.2 | 5.8 | 6 | 5.5 | **5.6** | 5.8 | 6.3 | 5.7 | 6.8 | 4.7 | **5.8** | 5.7 |
|  | **0.165** | 6 | 6.9 | 7 | 8.2 | 7.3 | **7.1** | 8.5 | 7.8 | 8.5 | 8.3 | 8.1 | **8.2** | 7.6 |
|  | **0.252** | 6.8 | 8.6 | 8.6 | 7.8 | 7.8 | **7.9** | 10.2 | 8.6 | 8.5 | 10.7 | 10.5 | **9.7** | 8.8 |
|  | **0.354** | 8.7 | 8.8 | 8.9 | 10.3 | 9.2 | **9.2** | 10.9 | 10.6 | 12.8 | 12.8 | 12.9 | **12** | 10.6 |
|  | **0.500** | 11 | 12.2 | 12.3 | 12.8 | 12.8 | **12.2** | 11.6 | 13.1 | 13.7 | 13.5 | 12.8 | **12.9** | 12.6 |
|  | **0.640** | 14 | 13.8 | 13.1 | 13.1 | 12.6 | **13.3** | 9.7 | 9.4 | 8.8 | 8 | 8.8 | **8.9** | 11.1 |
| **8** | **0.000** | 5.7 | 5.8 | 5 | 5.4 | 4.7 | **5.3** | 4.6 | 5.1 | 4.8 | 4.5 | 4.7 | **4.7** | 5 |
|  | **0.072** | 4.6 | 5.5 | 6.3 | 6.9 | 5.9 | **5.8** | 5.1 | 6.3 | 6 | 6.2 | 5.8 | **5.9** | 5.9 |
|  | **0.165** | 6.3 | 7.8 | 5.9 | 7.8 | 7.3 | **7.1** | 8 | 8.9 | 8 | 7.4 | 7 | **7.9** | 7.5 |
|  | **0.252** | 8.6 | 7.4 | 8.3 | 9 | 9 | **8.5** | 8.3 | 7.8 | 8.9 | 8.7 | 10.2 | **8.8** | 8.6 |
|  | **0.354** | 8.2 | 10.1 | 9.3 | 9.8 | 8.6 | **9.2** | 8.3 | 9.7 | 10.3 | 9.7 | 11.9 | **10** | 9.6 |
|  | **0.500** | 10.4 | 11.7 | 11.9 | 11.1 | 11.6 | **11.3** | 9.7 | 10.1 | 10.4 | 9.3 | 10.1 | **9.9** | 10.6 |
|  | **0.640** | 10.2 | 11.6 | 10.6 | 10.8 | 11.7 | **11** | 5.7 | 4.4 | 3.1 | 4.3 | 4.1 | **4.3** | 7.7 |
| **10** | **0.000** | 5.4 | 5 | 4.8 | 5.7 | 5.7 | **5.3** | 4.8 | 4.3 | 4.8 | 5.1 | 4.4 | **4.7** | 5 |
|  | **0.072** | 5.7 | 6.2 | 5.3 | 5.9 | 5.8 | **5.8** | 4.9 | 5.6 | 5.5 | 5.4 | 5.7 | **5.4** | 5.6 |
|  | **0.165** | 8.2 | 7.4 | 7 | 6.8 | 7.2 | **7.3** | 7.1 | 7.5 | 8 | 8.1 | 8.2 | **7.8** | 7.5 |
|  | **0.252** | 7.6 | 7.5 | 7.8 | 8.6 | 7.6 | **7.8** | 7.7 | 9.3 | 9.3 | 9.4 | 8.8 | **8.9** | 8.3 |
|  | **0.354** | 8 | 9.2 | 8.2 | 9.3 | 9.4 | **8.8** | 9.2 | 10.3 | 9.8 | 9.5 | 10.5 | **9.9** | 9.3 |
|  | **0.500** | 9.8 | 9.8 | 11.5 | 10.9 | 11.6 | **10.7** | 7.6 | 7.2 | 7 | 7.2 | 7.3 | **7.3** | 9 |
|  | **0.640** | 9.4 | 9.6 | 8.6 | 8.8 | 10 | **9.3** | 3.8 | 3.1 | 2.3 | 2.8 | 1.8 | **2.8** | 6 |
| **15** | **0.000** | 5.1 | 4.3 | 4.9 | 4.7 | 4.6 | **4.7** | 5.5 | 4.5 | 4.8 | 4.8 | 5.1 | **4.9** | 4.8 |
|  | **0.072** | 5.7 | 4.7 | 6.2 | 5.5 | 6.4 | **5.7** | 6.2 | 6.2 | 6.4 | 6.5 | 5.8 | **6.2** | 6 |
|  | **0.165** | 7.2 | 7.3 | 5.9 | 6.7 | 5.7 | **6.5** | 7.3 | 7.2 | 6.2 | 8.3 | 7.5 | **7.3** | 6.9 |
|  | **0.252** | 7.3 | 7.3 | 7.2 | 7.6 | 7.5 | **7.4** | 7.3 | 8.6 | 8.3 | 8.7 | 8.2 | **8.2** | 7.8 |
|  | **0.354** | 7.7 | 7.8 | 9.4 | 8.3 | 9.2 | **8.5** | 8.1 | 7 | 7.4 | 8 | 8.2 | **7.7** | 8.1 |
|  | **0.500** | 8.3 | 9.2 | 8.2 | 7.1 | 9.3 | **8.4** | 4.5 | 3.4 | 3.1 | 3 | 3 | **3.4** | 5.9 |
|  | **0.640** | 5.6 | 5.5 | 6.6 | 4.9 | 5.1 | **5.5** | 0.7 | 0.4 | 0.5 | 0.4 | 0.4 | **0.5** | 3 |
| **30** | **0.000** | 4.2 | 5 | 4.8 | 4.6 | 5.4 | **4.8** | 5.7 | 5.8 | 4.8 | 5.1 | 5.1 | **5.3** | 5 |
|  | **0.072** | 5.9 | 5.2 | 5.7 | 5 | 5.5 | **5.5** | 5.8 | 6.5 | 5.6 | 5.9 | 4.7 | **5.7** | 5.6 |
|  | **0.165** | 7 | 6.3 | 5.9 | 6.2 | 7.8 | **6.6** | 6.4 | 7.3 | 7.3 | 6.8 | 6.7 | **6.9** | 6.8 |
|  | **0.252** | 6.8 | 5.3 | 6.8 | 5.6 | 7 | **6.3** | 6.2 | 5.7 | 5.8 | 5.8 | 5.4 | **5.8** | 6 |
|  | **0.354** | 6.6 | 7 | 5.9 | 7.8 | 6.9 | **6.8** | 3.8 | 3 | 2.8 | 2.7 | 1.8 | **2.8** | 4.8 |
|  | **0.500** | 4.7 | 4.7 | 4.5 | 4.3 | 3.5 | **4.3** | 0.4 | 0.4 | 0 | 0.2 | 0 | **0.2** | 2.3 |
|  | **0.640** | 1.1 | 1.1 | 1 | 1.1 | 1.1 | **1.1** | 0 | 0 | 0 | 0 | 0 | **0** | 0.5 |
| **50** | **0.000** | 5 | 4.5 | 5.5 | 4.5 | 4.8 | **4.8** | 5.3 | 5.5 | 3.8 | 4.4 | 5.2 | **4.9** | 4.9 |
|  | **0.072** | 5.1 | 5.7 | 5.5 | 5.9 | 5.8 | **5.6** | 5.5 | 5.1 | 5.7 | 6 | 4.8 | **5.4** | 5.5 |
|  | **0.165** | 6.6 | 6.7 | 6.5 | 6.2 | 5.4 | **6.3** | 4.9 | 6.3 | 5.5 | 5.3 | 4.6 | **5.3** | 5.8 |
|  | **0.252** | 6.4 | 6.8 | 6.5 | 6.7 | 6.1 | **6.5** | 3.8 | 4.3 | 3.4 | 3.5 | 3.2 | **3.6** | 5.1 |
|  | **0.354** | 5.2 | 4.3 | 4.5 | 4.8 | 5.2 | **4.8** | 1.7 | 1 | 0.9 | 1 | 0.2 | **1** | 2.9 |
|  | **0.500** | 2.4 | 1.2 | 1 | 1.5 | 1.5 | **1.5** | 0 | 0 | 0 | 0 | 0 | **0** | 0.7 |
|  | **0.640** | 0 | 0.1 | 0 | 0 | 0 | **0** | 0 | 0 | 0.2 | 0.3 | 0 | **0.1** | 0.1 |
| **100** | **0.000** | 5.1 | 4.8 | 4.8 | 4.4 | 5.6 | **4.9** | 4.5 | 5.1 | 4 | 6.4 | 5 | **5** | 5 |
|  | **0.072** | 5.2 | 5 | 5.5 | 5.4 | 4.6 | **5.1** | 4.7 | 5.1 | 6.3 | 5 | 4.6 | **5.1** | 5.1 |
|  | **0.165** | 5.5 | 5.9 | 5 | 6.2 | 6.6 | **5.8** | 4.2 | 3.8 | 3.5 | 5.2 | 3.4 | **4** | 4.9 |
|  | **0.252** | 3.6 | 3.6 | 3.5 | 4.2 | 4 | **3.8** | 1.8 | 1.6 | 1.3 | 0.8 | 1.2 | **1.3** | 2.6 |
|  | **0.354** | 2.3 | 1.7 | 1.2 | 1.1 | 0.8 | **1.4** | 0 | 0 | 0 | 0 | 0 | **0** | 0.7 |
|  | **0.500** | 0.3 | 0 | 0.1 | 0 | 0.4 | **0.2** | 0 | 0 | 0 | 0 | 0 | **0** | 0.1 |
|  | **0.640** | 0 | 0 | 0 | 0 | 0 | **0** | 0 | 0 | 0 | 0 | 0 | **0** | 0 |

**Table S5: Percentages of datasets with no significant σ_int_^2^ in the full model (type II error when pICC>0) and type I error in the restricted model.**
