# Supplementary material for: When One Size Does Not Fit All: A Simple Statistical Method to Deal with Across-Individual Variations of Effects
Source: PLoS One. 2012 Jun 18;7(6):e39059. doi: 10.1371/journal.pone.0039059 (PMC3377596; doi:10.1371/journal.pone.0039059)
Supplement: Table S6 — Type I error rates (pICC = 0) and power (pICC>0) of UKS tests (%) for the same 490 designs as in Supplementary Table 1. Note that as for ME power (Table S3), the percentages increase with pICC and the number of individuals. (DOC) [file pone.0039059.s006.doc]

| **Nb Cond (*C*)** | | **2** | | | | | | **4** | | | | | |  |
| --- | --- | --- | --- | --- | --- | --- | --- | --- | --- | --- | --- | --- | --- | --- |
| **Nb Repet (*N*)** | | **3** | **5** | **10** | **20** | **40** | **Mean** | **3** | **5** | **10** | **20** | **40** | **Mean** | **GdMn** |
| **Nb Indv *I*** | **pICC** |  |  |  |  |  |  |  |  |  |  |  |  |  |
| **6** | **0.000** | 5 | 5 | 4 | 6 | 5 | **5** | 5 | 5 | 5 | 4 | 5 | **5** | 5 |
|  | **0.072** | 6 | 6 | 6 | 6 | 5 | **6** | 6 | 7 | 7 | 6 | 7 | **7** | 6 |
|  | **0.165** | 7 | 7 | 7 | 9 | 9 | **8** | 11 | 11 | 11 | 12 | 11 | **11** | 10 |
|  | **0.252** | 10 | 11 | 9 | 10 | 10 | **10** | 16 | 17 | 18 | 17 | 17 | **17** | 14 |
|  | **0.354** | 13 | 14 | 13 | 15 | 13 | **14** | 24 | 25 | 26 | 29 | 28 | **26** | 20 |
|  | **0.500** | 19 | 21 | 22 | 21 | 23 | **21** | 41 | 45 | 49 | 49 | 52 | **47** | 34 |
|  | **0.640** | 31 | 33 | 34 | 36 | 36 | **34** | 67 | 70 | 76 | 74 | 77 | **73** | 53 |
| **8** | **0.000** | 5 | 5 | 4 | 5 | 5 | **5** | 5 | 4 | 5 | 5 | 5 | **5** | 5 |
|  | **0.072** | 6 | 5 | 6 | 6 | 6 | **6** | 6 | 9 | 8 | 7 | 8 | **7** | 7 |
|  | **0.165** | 8 | 7 | 7 | 9 | 9 | **8** | 11 | 13 | 12 | 12 | 13 | **12** | 10 |
|  | **0.252** | 11 | 12 | 12 | 11 | 11 | **11** | 17 | 20 | 21 | 19 | 20 | **19** | 15 |
|  | **0.354** | 15 | 15 | 15 | 14 | 16 | **15** | 28 | 30 | 33 | 32 | 35 | **32** | 23 |
|  | **0.500** | 24 | 24 | 24 | 27 | 26 | **25** | 51 | 55 | 57 | 61 | 59 | **57** | 41 |
|  | **0.640** | 39 | 39 | 42 | 41 | 43 | **41** | 76 | 80 | 84 | 86 | 87 | **83** | 62 |
| **10** | **0.000** | 6 | 5 | 6 | 5 | 5 | **5** | 5 | 5 | 5 | 5 | 4 | **5** | 5 |
|  | **0.072** | 6 | 6 | 6 | 6 | 7 | **6** | 8 | 7 | 7 | 7 | 9 | **8** | 7 |
|  | **0.165** | 8 | 8 | 9 | 10 | 8 | **9** | 13 | 12 | 13 | 14 | 14 | **13** | 11 |
|  | **0.252** | 11 | 11 | 12 | 12 | 12 | **11** | 19 | 22 | 22 | 24 | 24 | **22** | 17 |
|  | **0.354** | 16 | 15 | 17 | 15 | 18 | **16** | 32 | 35 | 37 | 38 | 38 | **36** | 26 |
|  | **0.500** | 26 | 28 | 29 | 31 | 31 | **29** | 55 | 63 | 64 | 67 | 66 | **63** | 46 |
|  | **0.640** | 43 | 45 | 47 | 47 | 46 | **46** | 83 | 88 | 91 | 92 | 91 | **89** | 67 |
| **15** | **0.000** | 6 | 5 | 4 | 6 | 4 | **5** | 5 | 5 | 6 | 5 | 5 | **5** | 5 |
|  | **0.072** | 7 | 6 | 7 | 6 | 7 | **7** | 8 | 8 | 9 | 9 | 9 | **9** | 8 |
|  | **0.165** | 10 | 10 | 10 | 9 | 9 | **10** | 15 | 16 | 17 | 16 | 17 | **16** | 13 |
|  | **0.252** | 12 | 12 | 14 | 15 | 14 | **14** | 25 | 27 | 27 | 30 | 29 | **28** | 21 |
|  | **0.354** | 19 | 20 | 21 | 22 | 21 | **21** | 42 | 44 | 50 | 49 | 50 | **47** | 34 |
|  | **0.500** | 32 | 36 | 35 | 40 | 38 | **36** | 73 | 78 | 80 | 81 | 82 | **79** | 58 |
|  | **0.640** | 55 | 58 | 59 | 62 | 61 | **59** | 95 | 96 | 97 | 98 | 98 | **97** | 78 |
| **30** | **0.000** | 4 | 6 | 5 | 5 | 4 | **5** | 5 | 5 | 5 | 6 | 5 | **5** | 5 |
|  | **0.072** | 7 | 7 | 7 | 7 | 6 | **7** | 11 | 10 | 10 | 10 | 11 | **10** | 9 |
|  | **0.165** | 13 | 12 | 13 | 13 | 13 | **13** | 20 | 22 | 24 | 26 | 25 | **23** | 18 |
|  | **0.252** | 18 | 20 | 19 | 22 | 21 | **20** | 40 | 41 | 42 | 45 | 46 | **43** | 31 |
|  | **0.354** | 28 | 31 | 32 | 32 | 33 | **31** | 64 | 70 | 74 | 74 | 73 | **71** | 51 |
|  | **0.500** | 54 | 55 | 58 | 61 | 60 | **58** | 93 | 96 | 97 | 97 | 98 | **96** | 77 |
|  | **0.640** | 81 | 83 | 84 | 88 | 87 | **85** | 100 | 100 | 100 | 100 | 100 | **100** | 92 |
| **50** | **0.000** | 5 | 5 | 5 | 5 | 5 | **5** | 4 | 5 | 5 | 5 | 3 | **5** | 5 |
|  | **0.072** | 8 | 7 | 8 | 8 | 9 | **8** | 12 | 11 | 12 | 11 | 13 | **12** | 10 |
|  | **0.165** | 14 | 15 | 16 | 15 | 15 | **15** | 30 | 32 | 34 | 31 | 37 | **33** | 24 |
|  | **0.252** | 25 | 26 | 26 | 27 | 26 | **26** | 52 | 58 | 62 | 62 | 67 | **60** | 43 |
|  | **0.354** | 39 | 42 | 46 | 44 | 47 | **43** | 82 | 87 | 89 | 91 | 92 | **88** | 66 |
|  | **0.500** | 71 | 74 | 78 | 81 | 78 | **76** | 99 | 100 | 100 | 100 | 100 | **100** | 88 |
|  | **0.640** | 95 | 96 | 97 | 97 | 97 | **96** | 100 | 100 | 100 | 100 | 100 | **100** | 98 |
| **100** | **0.000** | 5 | 5 | 5 | 5 | 7 | **5** | 4 | 4 | 4 | 6 | 6 | **5** | 5 |
|  | **0.072** | 9 | 9 | 9 | 10 | 10 | **9** | 16 | 15 | 17 | 17 | 17 | **16** | 13 |
|  | **0.165** | 20 | 21 | 22 | 22 | 22 | **21** | 46 | 49 | 53 | 54 | 54 | **51** | 36 |
|  | **0.252** | 37 | 41 | 44 | 40 | 47 | **42** | 77 | 82 | 86 | 86 | 87 | **84** | 63 |
|  | **0.354** | 62 | 66 | 71 | 72 | 73 | **69** | 98 | 99 | 99 | 100 | 100 | **99** | 84 |
|  | **0.500** | 94 | 95 | 96 | 97 | 98 | **96** | 100 | 100 | 100 | 100 | 100 | **100** | 98 |
|  | **0.640** | 100 | 100 | 100 | 100 | 100 | **100** | 100 | 100 | 100 | 100 | 100 | **100** | 100 |

**Table S6: Type I error rates (pICC=0) and power (pICC>0) of UKS tests (%).**
