# Supplementary material for: When One Size Does Not Fit All: A Simple Statistical Method to Deal with Across-Individual Variations of Effects
Source: PLoS One. 2012 Jun 18;7(6):e39059. doi: 10.1371/journal.pone.0039059 (PMC3377596; doi:10.1371/journal.pone.0039059)
Supplement: Table S7 — Significance rates with UKS test minus significance rates for the random effect component of the full model in ME analyses, for the same 490 designs as in Table S1. The UKS test is more powerful than ME for evidencing non-null σint 2 component in designs with a small number of individuals or a low pICC. (DOC) [file pone.0039059.s007.doc]

| **Nb Cond (*C*)** | | **2** | | | | | | **4** | | | | | |  |
| --- | --- | --- | --- | --- | --- | --- | --- | --- | --- | --- | --- | --- | --- | --- |
| **Nb Repet (*N*)** | | **3** | **5** | **10** | **20** | **40** | **Mean** | **3** | **5** | **10** | **20** | **40** | **Mean** | **GdMn** |
| **Nb Indiv *I*** | **pICC** |  |  |  |  |  |  |  |  |  |  |  |  |  |
| **6** | **0.000** | 3 | 3 | 3 | 4 | 4 | **3** | 4 | 3 | 3 | 3 | 3 | **3** | 3 |
|  | **0.072** | 4 | 4 | 4 | 4 | 3 | **4** | 3 | 4 | 4 | 4 | 5 | **4** | 4 |
|  | **0.165** | 3 | 3 | 3 | 5 | 4 | **4** | 5 | 5 | 4 | 5 | 4 | **5** | 4 |
|  | **0.252** | 4 | 5 | 4 | 4 | 5 | **4** | 5 | 7 | 6 | 6 | 7 | **6** | 5 |
|  | **0.354** | 4 | 4 | 3 | 5 | 2 | **4** | 5 | 3 | 3 | 5 | 3 | **4** | 4 |
|  | **0.500** | 1 | 1 | 0 | 0 | 0 | **0** | 1 | 1 | 0 | 0 | 0 | **0** | 0 |
|  | **0.640** | -4 | -2 | -5 | -5 | -7 | **-4** | -3 | -3 | -3 | -6 | -3 | **-3** | -4 |
| **8** | **0.000** | 3 | 3 | 3 | 3 | 3 | **3** | 4 | 2 | 3 | 3 | 3 | **3** | 3 |
|  | **0.072** | 4 | 3 | 4 | 3 | 3 | **3** | 3 | 4 | 4 | 3 | 4 | **4** | 3 |
|  | **0.165** | 3 | 3 | 3 | 4 | 4 | **4** | 4 | 5 | 5 | 4 | 5 | **5** | 4 |
|  | **0.252** | 4 | 4 | 4 | 4 | 4 | **4** | 4 | 6 | 5 | 4 | 5 | **5** | 4 |
|  | **0.354** | 4 | 3 | 2 | 2 | 3 | **3** | 3 | 2 | 3 | 2 | 5 | **3** | 3 |
|  | **0.500** | 0 | -1 | -3 | -1 | -2 | **-2** | 0 | -3 | -3 | -3 | -3 | **-3** | -2 |
|  | **0.640** | -5 | -8 | -9 | -10 | -10 | **-8** | -6 | -7 | -6 | -4 | -4 | **-5** | -7 |
| **10** | **0.000** | 4 | 3 | 4 | 3 | 4 | **4** | 3 | 2 | 3 | 4 | 3 | **3** | 3 |
|  | **0.072** | 3 | 3 | 3 | 3 | 4 | **3** | 3 | 2 | 4 | 4 | 4 | **3** | 3 |
|  | **0.165** | 4 | 3 | 4 | 4 | 4 | **4** | 4 | 3 | 3 | 5 | 5 | **4** | 4 |
|  | **0.252** | 3 | 3 | 2 | 4 | 3 | **3** | 5 | 5 | 4 | 5 | 4 | **5** | 4 |
|  | **0.354** | 2 | 2 | 1 | 0 | 2 | **1** | 3 | 2 | 1 | 0 | 2 | **1** | 1 |
|  | **0.500** | -2 | -4 | -3 | -3 | -3 | **-3** | -3 | -3 | -5 | -5 | -6 | **-4** | -4 |
|  | **0.640** | -10 | -13 | -12 | -11 | -13 | **-12** | -5 | -4 | -4 | -4 | -5 | **-4** | -8 |
| **15** | **0.000** | 2 | 3 | 1 | 4 | 2 | **3** | 3 | 3 | 3 | 3 | 3 | **3** | 3 |
|  | **0.072** | 4 | 3 | 3 | 3 | 4 | **3** | 3 | 4 | 5 | 4 | 4 | **4** | 4 |
|  | **0.165** | 4 | 5 | 3 | 4 | 2 | **4** | 4 | 4 | 4 | 5 | 3 | **4** | 4 |
|  | **0.252** | 2 | 1 | 4 | 2 | 3 | **2** | 4 | 3 | 3 | 4 | 2 | **3** | 3 |
|  | **0.354** | -2 | -1 | 0 | -1 | 0 | **-1** | 0 | -3 | -2 | -2 | -2 | **-2** | -1 |
|  | **0.500** | -6 | -8 | -9 | -10 | -11 | **-9** | -6 | -7 | -7 | -7 | -5 | **-7** | -8 |
|  | **0.640** | -14 | -16 | -16 | -16 | -16 | **-16** | -3 | -3 | -1 | -2 | -1 | **-2** | -9 |
| **30** | **0.000** | 2 | 3 | 2 | 3 | 3 | **3** | 3 | 3 | 3 | 3 | 3 | **3** | 3 |
|  | **0.072** | 3 | 4 | 3 | 4 | 2 | **3** | 5 | 3 | 3 | 4 | 4 | **4** | 3 |
|  | **0.165** | 3 | 3 | 3 | 3 | 3 | **3** | 2 | 3 | 3 | 4 | 3 | **3** | 3 |
|  | **0.252** | 1 | 0 | -1 | 0 | -1 | **0** | 1 | -1 | -4 | -2 | 0 | **-1** | -1 |
|  | **0.354** | -5 | -6 | -9 | -7 | -7 | **-7** | -5 | -7 | -8 | -9 | -9 | **-8** | -7 |
|  | **0.500** | -14 | -15 | -15 | -13 | -15 | **-14** | -4 | -3 | -2 | -2 | -2 | **-3** | -8 |
|  | **0.640** | -13 | -12 | -12 | -9 | -9 | **-11** | 0 | 0 | 0 | 0 | 0 | **0** | -6 |
| **50** | **0.000** | 3 | 3 | 3 | 3 | 3 | **3** | 2 | 3 | 3 | 2 | 2 | **2** | 2 |
|  | **0.072** | 3 | 3 | 3 | 3 | 3 | **3** | 4 | 3 | 3 | 4 | 5 | **4** | 3 |
|  | **0.165** | 2 | 2 | 2 | 2 | 1 | **2** | 3 | 2 | 2 | 0 | -1 | **1** | 1 |
|  | **0.252** | -2 | -1 | -5 | -5 | -4 | **-3** | -5 | -6 | -4 | -5 | -7 | **-5** | -4 |
|  | **0.354** | -12 | -13 | -13 | -14 | -12 | **-13** | -8 | -5 | -5 | -5 | -4 | **-5** | -9 |
|  | **0.500** | -15 | -15 | -13 | -12 | -14 | **-14** | -1 | 0 | 0 | 0 | 1 | **0** | -7 |
|  | **0.640** | -4 | -4 | -3 | -3 | -3 | **-3** | 0 | 0 | 1 | 1 | 0 | **0** | -2 |
| **100** | **0.000** | 3 | 3 | 3 | 3 | 3 | **3** | 2 | 2 | 3 | 4 | 5 | **3** | 3 |
|  | **0.072** | 2 | 3 | 2 | 3 | 3 | **2** | 5 | 4 | 5 | 2 | 2 | **4** | 3 |
|  | **0.165** | -2 | 0 | -2 | -3 | -4 | **-2** | -2 | -3 | -4 | -5 | -5 | **-4** | -3 |
|  | **0.252** | -9 | -10 | -10 | -10 | -7 | **-9** | -8 | -7 | -6 | -5 | -6 | **-7** | -8 |
|  | **0.354** | -19 | -17 | -15 | -14 | -18 | **-16** | -2 | -1 | -1 | 0 | 0 | **-1** | -9 |
|  | **0.500** | -5 | -5 | -4 | -3 | -1 | **-4** | 0 | 0 | 0 | 0 | 0 | **0** | -2 |
|  | **0.640** | 0 | 0 | 0 | 0 | 1 | **0** | 0 | 0 | 0 | 0 | 0 | 0 | 0 |

**Table S7: Significance rates with UKS test minus significance rates for the random effect component of the full model in ME analyses.**
