# Supplementary material for: When One Size Does Not Fit All: A Simple Statistical Method to Deal with Across-Individual Variations of Effects
Source: PLoS One. 2012 Jun 18;7(6):e39059. doi: 10.1371/journal.pone.0039059 (PMC3377596; doi:10.1371/journal.pone.0039059)
Supplement: Results S1 — This section provides further results on the UKS test and additional detail on type II errors as a function of inter- and intra-individual variances (1), type I errors with violations of homoscedasticity in individual Anovas (2), type I errors with violations of normality in individual one-way Anovas (3), and Mixed-effects models analyses (4). (DOC) [file pone.0039059.s008.doc]

**1. Type II errors as a function of inter- and intra-individual variances**

The right part of Panel D in Figure 1 shows that the iso-probability lines of the UKS test procedures asymptote to lines converging towards the point of coordinates (–0.5, 0). This is a general feature that would be observed for any design. Indeed, for individual Anovas, the expected values for the individual F denominator and numerator are $\text{E}(Num)=S_{\text{eff}}+\text{σ}_{\mathrm{int}}^{2}+\text{σ}_{err}^{2}/N$ and $\text{E}(Den)=\text{σ}_{\text{err}}^{2}/N$. Considering their ratio *R* as a first-order approximation of the expected F value, we can write that $\text{σ}_{\text{err}}^{2}/N=(S_{\text{eff}}+\text{σ}_{\mathrm{int}}^{2})/(R-1)$. When σ_int_^2^ is large with respect to *S*_eff_, $\text{σ}_{\text{err}}/\sqrt{N}\cong(\sigma_{\mathrm{int}}+S_{\text{eff}}/2)/\sqrt{(R-1)}$. This is the equation of a line with slope 1/√(*R*-1) crossing the X axis at the coordinate –*S*_eff_/2. The reason why the asymptotes of UKS test iso-probability curves correspond to constant expected individual F values is that similar individual F distributions yield similar UKS test results and similar expected F values.

We checked the later statement by analyzing the distribution of individual F for 3 series of 10 to 15 (σ_err_, σ_int_) couples close to the part of the 0.05, 0.01 and 0.001 curves with σ_int_ ≥ 3. We find that the average of individual F (3.26, 5.23 and 9.09 for the 0.05, 0.01 and 0.001 curves, respectively) were close to estimations of *R* (3.25, 5.34 and 9.65) derived from empirical measures of the asymptotes slopes (0.66, 0.48 and 0.34). In addition, the fact that both the median and mean of individual F values were highly consistent across (σ_err_, σ_int_) couples along the same curve confirmed that individual F distributions were similar.

To shed more light on how the test works, we further analyzed simulation data. For the .05 threshold, with σ_int_ ≥ 3, the median F and *p*-value amounts to 1.38 and 0.271. The UKS test is significant at the .05 threshold (T_K_ < 0.369) if there are 4 individual *p*-values below .031 (4/10 - 0.369), or 5 below .131, or 6 below .231 and so on up to 10 values below .631. We found that the maximal value for T_K_ was seldom reach with 4 *p*-values below the smaller threshold (2% of significant T_K_) or 10 *p*-values below the higher threshold (6%), but generally with 6 to 8 *p*-values below their respective threshold (65%). For the .01 threshold (median *p*-value 0.174; smallest threshold 0.043 for 5 *p*-values) and the .001 threshold (median *p*-value 0.082; smallest threshold 0.045 for 6 *p*-values), the maximal T_K_ value was also reached with medium rather than minimal or maximal number of individual *p*-values. This showed that with realistic distributions of inter-individual differences in effect value and trial-to-trial errors, the results of the UKS test rely on a majority of individual tests rather than on few of them or all of them.

**2. Type I errors with violations of homoscedasticity in individual Anovas**

We analyzed the distribution of individual *p*-values to get insight on how the excess of type I errors in individual Anovas with heteroscedasticity was watered down by the UKS test. We found that the individual *p*-value density was higher-than-normal from 0 to 0.10 and from 0.75 to 1, and lower-than-normal from 0.10 to 0.75. As a consequence, the difference between the cumulative distributions of individual Anovas probabilities and the uniform distribution had a relatively small maximum near 0.10 and became negative before 0.5, except for a slower decrease in Anovas with 2-levels factor. We conjectured that for the UKS test the excess of individual *p*-values in the 0-0.1 range was compensated by the deficit in the 0.1-0.5 range.

In a second additional analysis, we assessed whether the Levene’s and Bartlett’s tests of unequal variances could help determining when the UKS test can be used safely. We found these tests quite useless for being more sensitive to sample size than to heteroscedasticity. For example, with the highest variance ratio (8) the proportion of significant individual datasets with 5 within-level repetitions did not exceed 20 % (Levene's) or 35 % (Bartlett's), whereas with the lowest variance ratio both tests evidenced unequal variances in 50 % of individual datasets with 40 within-level repetitions.

**3. Type I errors with violations of normality in individual one-way Anovas**

As for heteroscedasticity, we analyzed the distribution of individual *p*-values to get insight on how skewness in individual data inflated type I errors in UKS test. We found that the bias in the distribution of individual Anovas probabilities is opposite to the bias evidenced for heteroscedasticity. When the factor has no effect, the density of probabilities is abnormally low in the 0-0.15 range, but abnormally high in the 0.15-0.70 range. Accordingly, the one-sample KS test is biased towards finding that the proportion of probabilities in the 0.15-0.70 range is significantly higher than in a [0 1] uniform distribution. In keeping with the inter-design variations of the UKS test type I error rate, the bias in the distribution of individual Anovas *p*-value decreases as the number of condition levels increases, and tends to be highest for 5 to 10 repetitions.

Finally, all three analyses of simulation data about type I and type II errors highlight the importance of the individual *p*-values in the 0.1-0.5 range for the significance of the UKS test. In the type II error study at the .05 threshold, the maximal value of the UKS test is reached in 65 % of cases when there are 6 *p*-values below .231, or 7 below .331, or 8 below .441. When there is heteroscedasticity in individual data, the excess of individual *p*-values in the 0-0.1 range was compensated by the deficit in the 0.1-0.5 range. When individual data is skewed, the excess of individual *p*-values in the 0.15-0.70 range is amplified by the UKS test. It may be conjectured that in general violations of individual tests assumptions are more likely to affect the UKS test type I error rates when they increase the proportion of individual *p*-values in the 0.1-0.5 range than in the 0-0.1 range.

**4. Mixed-model analyses**

*General results.*

We found that the values and CI of all μ and σ parameters *but* σ_int_^2^ were generally accurately estimated. The estimates of the factor effect were accurate for any population size, often exact for null pICC or within 5% of their expected value for large pICC. The estimates of the error variance (σ_err_^2^), and thus the CI of the factor effect, were within 1% of their theoretical values for the full model when the number of repetitions exceeded 10 or the number of individuals exceeded 30. When datasets were analyzed according to the restricted model, the error variance was as expected overestimated by 1 to 7% as pICC increased from 0.16 to 0.64. The estimates of the intercept variance (σ_subj_^2^) were accurate when the number of individuals exceeded 50 but 2 to 10% too small as population size decreased from 30 to 6. When the population size criteria were met and the CIs available, σ_err_^2^ and σ_subj_^2^ CIs included the theoretical variances in 95% of cases, and their size, defined as the geometrical average of the ratio between upper and lower limits, perfectly matched the ratio of the .975 to .025 quantile of the random samples variances. For smaller population sizes, the CI size were no more than 2% too small for σ_err_^2^ in the full model and up to 15% too small for σ_subj_^2^, and the percentage of CI including the theoretical value did not decrease below 93 % (σ_err_^2^ in the full model) or 90% (σ_subj_^2^). Note that with the restricted model, the percentage of CI including the theoretical value varied from 95% to 0 % as the pICC and the number of individuals increased and the number of repetition decreased.

*Sources of low power for detecting significant* σ_int_^2^ *in the full model.*

The main problem concerned the estimation of the effect variance CI and consequently the power for evidencing non-null σ_int_^2^ when the pICC was low or when the number of individuals was small. In these situations, there were two related difficulties. First, the estimation of the CI frequently failed (Table S1). When the pICC was null or very low, the failure rate exceeded 50% for large numbers of individuals and repetitions. When the number of individuals was low (e.g. 10), the failure rate was around 10 % for pICC corresponding to common situations (e.g. pICC=0.5 for 5 repetitions with ICC equal to 0.17 or 10 repetitions with ICC=0.1). Second, when the CI could be estimated, it was often abnormally large. We assessed CI size by comparing the ratio of the upper to the lower CI limit to the ratio of the .975 to .025 quantile of the random sample variances. While for high ICC and population size the two ratios were equal, the first ratio could be several orders of magnitude greater than the second one for small ICC or population sizes. The classes of datasets with the largest proportion of ill-defined confidence intervals were the same that underwent the highest rates of failure in estimating CI (see Table S2 for the percentage of datasets with an upper to lower CI limit ratio above 100 times the ratio of the corresponding quantiles).

**Table legends**

**Table S1.**

The average percentage of failure in estimating the confidence intervals of variances using the R function *intervals* are displayed for 490 RM Anova designs. Each design is characterized by a number of individuals (6 to 100, column 1), a pICC value (0 to 0.64, column 2), a number of factor levels (2 or 4, top line) and a number of within-level repetitions (3 to 40, second line). The last column displays the grand mean across numbers of factor’s levels and repetitions. Averages are computed across 2000 datasets for most designs, and 500 or 1000 for the largest ones.

**Table S2.**

Percentages of datasets with ill-defined confidence intervals for the factor effect variance σ_int_^2^, for the same 490 designs as in Table S1. Ill-defined confidence intervals are arbitrarily defined as CI with ratio of upper to lower limit over 100 times the ratio of the .975 to .025 quantile of random sample variances. Detailed investigations showed that percentages steadily increase as the ratio threshold decrease from 100 to 1 and decrease as the ratio threshold increase from 100 to the computer-dependent maximal value. Thus, the present pattern of results does not depend on the choice of a particular ratio threshold.

**Table S3.**

The average percentage of datasets where a significantly non-null variance component σ_int_^2^ was evidenced are displayed for the same 490 RM Anova designs as in Table S1. For pICC equal to zero, the figures indicate the type I error rates, which are abnormally low (2 % instead of 5 %). For pICC above zero, the figures indicate the power.

**Table S4.**

Type I error rates (%) in the restricted model for the same 490 designs as in Table S1. These percentages concern all datasets, rather than only those with type II errors in the full model. Note that the percentages vary as a function of pICC and number of condition levels and do not depend on the number of individuals.

**Table S5.**

Percentages of datasets with no significant σ_int_^2^ in the full model (type II error when pICC>0) and type I error in the restricted model, for the same 490 designs as in Table S1. Note that for a low number of individuals (< 15), the percentages increase with pICC, thus with ICC and the number of repetitions.

**Table S6.**

Type I error rates (pICC=0) and power (pICC>0) of UKS tests (%) for the same 490 designs as in Supplementary Table 1. Note that as for ME power (Table S3), the percentages increase with pICC and the number of individuals.

**Table S7.**

Significance rates with UKS test minus significance rates for the random effect component of the full model in ME analyses, for the same 490 designs as in Table S1. The UKS test is more powerful than ME for evidencing non-null σ_int_^2^ component in designs with a small number of individuals or a low pICC.
